# Cytokinin‐responsive P‐type cyclins control Arabidopsis radial style morphology

**DOI:** 10.1111/tpj.70592

**Published:** 2025-11-24

**Authors:** Iqra Jamil, Samuel W. H. Koh, Jitender Cheema, Laila Moubayidin

**Affiliations:** ^1^ Department of Cell and Developmental Biology John Innes Centre Colney Lane NR4 7UH Norwich Norfolk UK; ^2^ Department of Computational and Systems Biology John Innes Centre Colney Lane NR4 7UH Norwich Norfolk UK; ^3^ Department of Chemistry Life Sciences and Environmental Sustainability, University of Parma 43124 Parma Italy; ^4^ Present address: Laboratory of Biochemistry Wageningen University Stippeneng 4 Wageningen 6708WE the Netherlands; ^5^ Present address: European Molecular Biology Laboratory European Bioinformatics Institute, Wellcome Genome Campus Hinxton Cambridge Cambridgeshire CB10 1SD UK

**Keywords:** style development, radial symmetry, cytokinin, CYCLIN‐P3s, gynoecium, transcriptional regulation, *Arabidopsis thaliana*

## Abstract

The biological mechanisms responsible for correct shape acquisition at the apex of the female reproductive organ, the gynoecium, remain poorly understood, despite its fundamental importance for successful plant reproduction and seed production. This process involves a rare bilateral‐to‐radial symmetry transition in *Arabidopsis thaliana,* orchestrated in part by the transcription factor SPATULA (SPT). Here, we show that SPT negatively regulates cell cycle regulators CYCLIN‐P3;1 (CYCP3;1) and CYCP3;2, which are antagonistically promoted by the hormone cytokinin (CK), to control the radial style morphology by orchestrating a coherent feed‐forward loop that converges on the spatial regulation of *CYCP3;1* and *CYCP3;2* expression. Overexpression of CYCP3s disrupts style radial symmetry, causing the split‐style phenotype and hypersensitivity to CK observed in the *spt* mutant. Finally, we demonstrate a genetic link connecting the machinery of cell division orientation, controlled by auxin, with the cell proliferation input induced by CK, which adds robustness to the apical fusion of the carpels.

## Significance Statement

The master regulator SPATULA (SPT) suppresses cytokinin (CK)‐induced cell proliferation to ensure carpel fusion, acting through a coherent feed‐forward loop (FFL) that converges on the cell cycle regulators CYCLIN‐P3;1 (CYCP3;1) and CYCP3;2. By fine‐tuning CK‐driven proliferation signals, SPT enhances the robustness of auxin‐controlled cell division orientation, thereby enabling proper style patterning and shape acquisition.

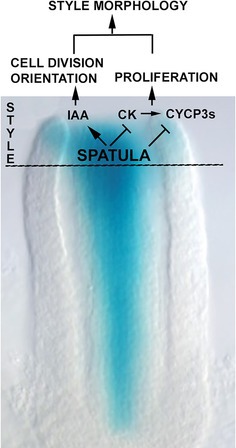

The master regulator SPT represses the CK‐driven cell proliferation input to ensure carpel fusion, by orchestrating a coherent FFL converging on the cell cycle regulators CYCLIN‐P3;1 (CYCP3;1) and CYCP3;2. Fine‐tuning the proliferation signals driven by CK enhances robustness in cell division orientation, enabling proper patterning and shape acquisition of the style.

## INTRODUCTION

Establishing the appropriate symmetry types, for example radial, biradial or bilateral symmetry, alongside specification of the polarity axes and tissue proliferation poses a significant challenge during the morphogenesis of plant and animal organs. In *Arabidopsis thaliana*, the bHLH transcription factor SPATULA (SPT) (Heisler et al., 2001) plays a crucial role in promoting the development of the apical part of the female reproductive organ, the gynoecium, by supporting patterning and tissue specification along the medial and adaxial body axis directions to form the radially symmetric style (Carabelli et al., 2021; Larsson et al., 2014; Moubayidin & Ostergaard, 2014).

**Figure 1:**
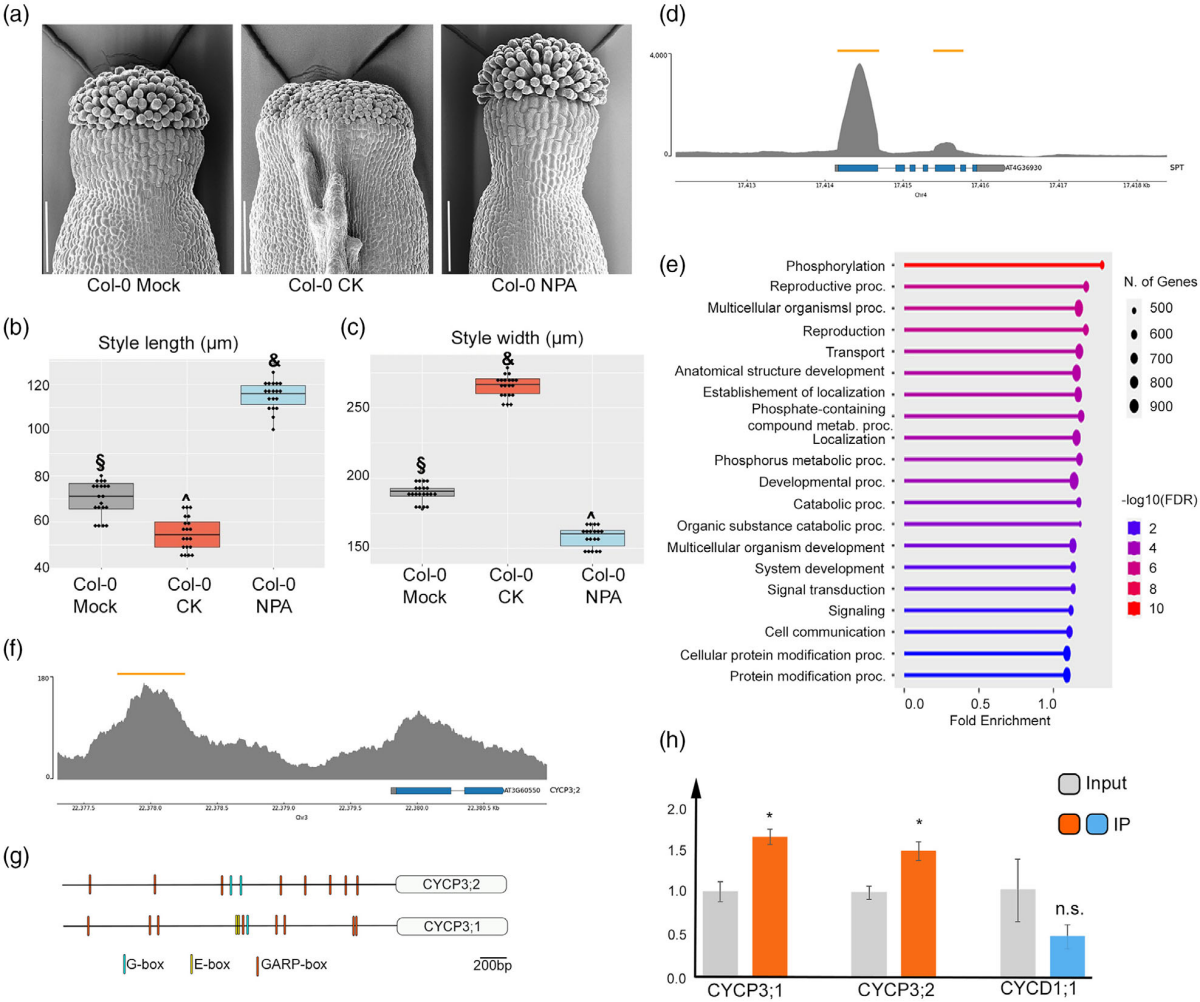
SPATULA (SPT) direct targets in Arabidopsis inflorescences include *CYCP3;1* and *CYCP3;2*. (a) Scanning electron microscope (SEM) images of wild‐type Col‐0 styles treated with mock (left panel), cytokinin (CK) (middle panel, 50 μM BAP) and naphthylphthalamic acid (NPA) (right panel, 100 μM). Scale bars represent 100 μm. (b, c) Boxplots of quantification of style length (b) and width (c) of samples depicted in (a). *n* = 3 biological replicates; results of one representative biological replicate are plotted. Dots on boxes represent number of samples analysed per treatment. Symbols (&, §, ^) on boxplots represent results of one‐way anova followed by Tukey's Honestly Significance Difference (HSD) test. Tukey's HSD *P* values for Col‐0 mock versus Col‐0 CK, Col‐0 mock versus Col‐0 NPA and Col‐0 CK versus Col‐0 NPA for both style length and width are <0.001 (&, §, ^). (d, f) Representative raw chromatin immunoprecipitation sequencing (ChIP‐seq) peaks of control gene *SPT* and an Arabidopsis P‐type cyclin gene, that is *CYCLIN‐P3;2* (*CYCP3;2*). *n* = 3 biological replicates; peaks of one representative replicate are shown. Yellow bars on top represent peaks position on chromosome. Blue bars on bottom show exons, and grey lines show introns. (e) Bar chart of Gene Ontology (GO) terms of biological processes enriched in the set of 6731 presumptive direct targets of SPT. ShinyGO (v0.80) was used to perform GO terms with the false discovery rate cut‐off 0.05. (g) Schematic representation of conserved motifs in the 5′ promoter regions of *CYCP3;1* and *CYCP3;2*. Different coloured bars on promoters represent binding sites for bHLH (G‐box in cyan and E‐box in yellow) and ARR‐Bs transcription factors (GARP‐box in orange). Note the presence of E‐box variant only in the promoter region of CYCP3;1. (h) Bar chart of ChIP‐qPCR showing enrichment levels of *CYCP3;1* and *CYCP3;2* and *CYCD1;1* in anti‐GFP antibody pull‐down immunoprecipitated (IP) samples compared to control (input) of *spt‐12*/*SPT::SPT:sYFP* inflorescences. The expression levels were normalised against *ACTIN7*. Error bars represent SD; *P < 0.05; ns signifies not statistically significant (unpaired Student's *t*‐test). *n* = 3 biological replicates.

The development of the cylindrical style structure (Figure 1a) requires dynamic control of auxin distribution via biosynthesis (Stepanova et al., 2008; Trigueros et al., 2009), signalling (Martinez‐Fernandez et al., 2014; Simonini et al., 2016) and transport (Moubayidin & Ostergaard, 2014). SPT functions are instrumental during the apical fusion of the two carpels by directing auxin accumulation at the gynoecium apex (Carabelli et al., 2021; Moubayidin & Ostergaard, 2014). We recently showed that the SPT‐mediated auxin accumulation at the medial‐apical cells is required to maintain the orientation of cell division in the periclinal direction, that is perpendicular to the apical‐basal direction of organ growth (Tasker‐Brown et al., 2024). This is in line with the documented anisotropic growth at the style (Eldridge et al., 2016; Gomez‐Felipe et al., 2024). Growth analysis studies of the Arabidopsis style highlighted that growth is largely orchestrated along the apical‐basal body axis. Accordingly, two upstream regulators of SPT, the *O*‐glycosyl transferases SECRET AGENT (SEC) and SPINDLY (SPY) have been recently shown to promote style elongation and cellular expansion (Jiang et al., 2024), consistent with a role for SPT in the local adjustment of cell division and growth.

Additionally, SPT is involved in a complex interplay that coordinates the morphological auxin signals with the proliferative signal mediated by the hormone cytokinin (CK) (Nemhauser et al., 2000; Ramos‐Pulido & de Folter, 2025; Reyes‐Olalde, Zuniga‐Mayo, Marsch‐Martinez, & de Folter, 2017; Schuster et al., 2015) which plays antagonistic roles to auxin throughout plant development, including gynoecium patterning (Moubayidin et al., 2009; Muller et al., 2017). CK applications to young gynoecia induce ectopic proliferation of the medial tissue and an increase of the CK signalling reporter *TCSn::GFP* is observed following 6‐Benzylaminopurine (BAP) treatments, in those ectopic growths as well as in the apical style (Ramos‐Pulido & de Folter, 2025). Accordingly, *spt* mutant styles are hypersensitive to CK applications, that is an increase in the frequency and severity of split styles is observed following BAP treatments (Reyes‐Olalde, Zuniga‐Mayo, Serwatowska, et al., 2017; Zuniga‐Mayo et al., 2014).

Despite the apical fusion of the gynoecium playing a pivotal role in ensuring efficient fertilisation and seed production, the molecular and cellular mechanisms that guide carpel fusion at the apical style remain elusive. Furthermore, several downstream activities of the transcription factor SPT, which are crucial for style radialisation, have yet to be elucidated. Given that SPT functions as a master regulator of carpel fusion, it likely governs multiple molecular frameworks. Consequently, the coordination of several pathways under direct SPT regulation may be required for proper style formation.

Among the diverse pathways regulated by SPT, the molecular framework linking SPT to CK‐induced proliferative input remains particularly unclear.

To gain deeper insight into the range of activities controlled by SPT, we performed a chromatin immunoprecipitation (ChIP)‐seq experiment using inflorescences from a functional complementation line (*spt‐12/SPT::SPT–sYFP*) (Jiang et al., 2024). This approach enabled the identification of 6731 genes associated with key developmental and cellular processes, including regulators of cell division. Coordination of proliferation and cell division orientation has been proposed to facilitate division plane determination in proliferating tissues, in the root meristem (Costa, 2017; Zhang et al., 2016).

This scenario is consistent with a requirement for a tight regulation of cell division activities during the apical fusion of the carpels where we hypothesised a robust placement of cell division might underpin the final fusion of the apical‐medial marginal tissue (Tasker‐Brown et al., 2024), where an auxin maximum picks and CK signalling response is inhibited.

Combining genetic, molecular and pharmacological experiments, we show that SPT directly and cell‐autonomously represses the expression of *CYCLIN‐P3;1* (*CYCP3;1*) and *CYCP3;2* (hereafter together referred to as CYCP3s) (Torres Acosta et al., 2004; Wang et al., 2004), specifically at the style, two poorly investigated cyclins that regulate meristematic cell division at the Arabidopsis root apical meristem (Chen et al., 2020). SPT represses both *CYCP3s* in an opposite fashion to the cell proliferation signal provided by CKs, which induces their expression. Our data demonstrate that CYCP3s' activity directly contributes to the bilateral style phenotype observed in the *spt* mutant, supporting their functional role in maintaining radial symmetry. Overexpressing CYCP3s lines displayed a significant number of unfused styles, resembling the *spt* split‐style phenotype. Moreover, radial styles were partially restored in the *spt cycp3;1 cycp3;2* triple loss‐of‐function mutant, and hypersensitivity to CK was reduced, meaning CYCP3s might play a role in controlling cell proliferation via the CK signalling pathway. Furthermore, we showed that combining defects in cell division orientation (by using loss‐of‐function mutants in key players involved with preprophase band (PPB) function/assembly) with perturbation in proliferation (by a tissue‐specific expression of a CK biosynthetic gene and via external CK applications), drastically impacts the apical fusion of the two carpels, that is break of style radial symmetry. Thus, SPT regulates style morphogenesis by, at least partially, working cell‐autonomously at the apical style cells, by simultaneously coordinating an auxin‐centric incoherent feed‐forward loop (FFL) (Tasker‐Brown et al., 2024) with a CK‐centric coherent feed‐forward regulatory loop, involving CYCP3s, to add robustness to style morphogenesis.

## RESULTS

### *In vivo* regulation of gene expression by SPT

To reveal the genes and processes regulated by SPT in the apical style region of the Arabidopsis gynoecium, we performed ChIP followed by deep sequencing (Seq) using inflorescence material from the SPT complementation line (*spt‐12/SPT*::*SPT*–*sYFP*) (Jiang et al., 2024).

We selected a list of 6731 high‐confidence candidate genes that consistently enriched in all three biological replicates using 0.001 false discovery rate (FDR) for peak calling.

By comparing the 3692 genes transcriptionally regulated by SPT, identified through RNA‐seq analysis of *35S::SPT* overexpressing seedlings (Bernal‐Gallardo et al., 2023), with our ChIP‐seq dataset, we identified 657 genes that are likely direct transcriptional targets of SPT, as they are both transcriptionally regulated by SPT and its putative direct targets (Table S1). Notably, SPT binds to its own promoter (Figure 1d; Table S1), a finding that provides a molecular explanation for the elevated *SPT* transcript levels previously observed in an SPT overexpressing line (Bernal‐Gallardo et al., 2023) and in our *spt‐12/SPT*::*SPT*–*sYFP* complementation line (Jiang et al., 2024).

As further positive controls, we examined genes previously reported to interact with SPT genetically or functionally during gynoecium development, such as the bHLH TFs INDEHISCENT (IND) (Girin et al., 2011) and HECATEs (HECs) (Gremski et al., 2007; Schuster et al., 2015), as well as CUP‐COTYLEDON 2 (CUC2) (Nahar et al., 2012) (Figure S1a–d; Table S1). These genes were found to be bound by SPT in all three biological replicates, supporting the established model that these transcription factors function coordinatively (Ballester et al., 2021).

To understand the biological processes controlled downstream of SPT, we analysed the top 20 Gene Ontology (GO) categories that were statistically enriched by ranking all 6731 presumptive SPT direct target genes. We found the processes related to reproduction, development and multicellular organismal processes were significantly enriched, notably including key regulators of style morphogenesis genetically linked to SPT, such as *SPT* itself, *IND, HEC1,2* and *CUC2* (Figure 1e; Table S1).

Other enriched GO terms highlighted roles for SPT in phosphorylation and phosphate‐related metabolic processes, signal transduction, transport, catabolism and protein modification processes (Figure 1e). These findings are consistent with previously identified roles of SPT in sugar‐based post‐translational modifications (Jiang et al., 2024), as well as auxin and CK signalling and transport (Marsch‐Martinez & de Folter, 2016; Nemhauser et al., 2000; Schuster et al., 2015).

The role of SPT in style radialisation has recently been linked to the regulation of cell division rate and orientation, specifically through the regulation of members of the D‐type cyclin family (CYCDs), namely CYCD1;1 and CYCD3;3 (Tasker‐Brown et al., 2024). Accordingly, we found enrichment of CYCD3;3 in two of the three biological replicates analysed (Figure S1e). However, neither loss‐of‐function nor overexpressing mutants of CYCDs have been reported to affect style morphogenesis on their own, suggesting that SPT regulates multiple aspects of cell division control to ensure correct style morphogenesis.

To untangle the underlying mechanisms involved in style morphology, we focused on presumptive downstream targets of SPT involved in cell cycle and cell division regulation (Table S1). A previous study showed that among the cell cycle‐related genes regulated by IND, while CYCD1;1 was upregulated, a member of the P‐type cyclin family (CYCPs), CYCP3;1, was found to be downregulated by IND (Simonini et al., 2016). In addition, CYCP3;1 was also found to be downregulated by SPT in seedlings (Table S1) (Bernal‐Gallardo et al., 2023) and transcriptionally regulated by B‐type CK response regulators in the shoot apical meristem (Xie et al., 2018). Interestingly, among the cell cycle genes downstream of SPT in our ChIP‐seq experiment (Table S1) CYCP3;1 sister protein, *CYCP3;2*, was found to be a high‐confidence candidate target of SPT in all three biological replicates with a nearest peak summit at 1.58 kb upstream of the transcription start site and within 200 bp vicinity of the core hexanucleotide sequence, that is G‐box (CACGTG) previously reported to be bound by SPT (Toledo‐Ortiz et al., 2003) (Figure 1f).

In Arabidopsis, CYCP3;2 and CYCP3;1, have been recently proposed as positive regulators of cell proliferation working from the root epidermis in an opposite fashion to Brassinosteroids (Chen et al., 2020; Torres Acosta et al., 2004), although their roles in development and cell cycle control remain largely unknown.

According to the presence of G‐box elements in the promoter of both *CYCP3s* (Figure 1g), we confirmed that both *CYCP3s* are direct targets of SPT by performing qPCR experiments from three independent biological ChIP materials of *spt‐12/SPT*::*SPT*–*sYFP* inflorescences (Figure 1h). Our experiments also showed that another cyclin genetically linked to SPT and IND, CYCD1;1 (Tasker‐Brown et al., 2024), is not a direct target of SPT (Figure 1h).

Altogether, our experiments shed light on the broader cellular and molecular roles of SPT activity in the inflorescence tissues, supporting the notion that SPT finetunes and coordinates multiple aspects of developmental cellular signalling, including hormonal responses and cell cycle regulation. Notably, our results also confirm that the two cell cycle regulators, *CYCP3;1* and *CYCP3;2*, are direct transcriptional targets of SPT.

### SPT represses *CYCP3s* expression in a coherent FFL with CKs

To investigate the causative molecular mechanisms linking cell division defects to the bilateral style phenotype in the *spt* mutant, we further characterised the roles of CYCP3s during style development. SPT has been reported to work as a growth repressor in roots and cotyledons (Ichihashi et al., 2010; Makkena & Lamb, 2013a, 2013b). Accordingly, in the style, SPT represses the CK output (Schuster et al., 2015), which has a proliferative effect on the abaxial marginal gynoecium tissues (Reyes‐Olalde, Zuniga‐Mayo, Serwatowska, et al., 2017) (Figure 1a). To pattern the style, SPT finetunes the auxin/CK crosstalk which has opposite effects on style development (Reyes‐Olalde, Zuniga‐Mayo, Marsch‐Martinez, & de Folter, 2017): while blocking auxin transport by naphthylphthalamic acid (NPA) applications leads to a thinner and longer wild‐type style without inducing ectopic growth, CK applications result in a wider and shorter style and promote ectopic proliferation (Figure 1a–c). Accordingly, *spt* bilateral style frequency and complexity are highly increased by CK applications (Schuster et al., 2015), strongly suggesting that SPT may control style morphology by repressing proliferation in an opposite manner to CK.

Because CYCP3s were also found among the downstream targets of the CK response regulators in the shoot apical meristem (Liu et al., 2020), we hypothesised that CYCP3s could be transcriptionally regulated by SPT and CK in an antagonistic way, that is downregulated by SPT and upregulated by CK in the style region.

**Figure 2:**
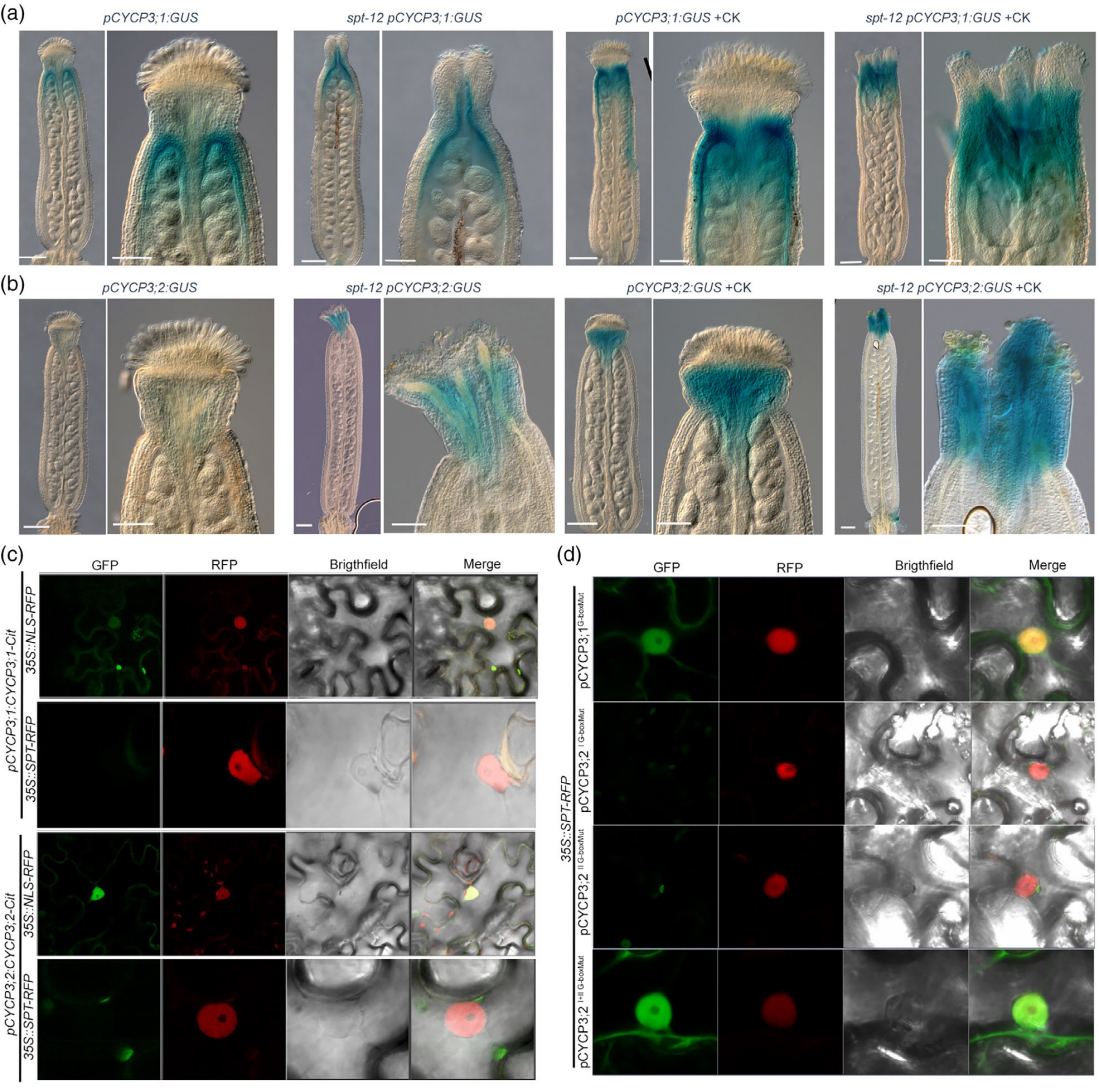
SPATULA (SPT) and cytokinin (CK) regulate *CYCP3s* expression in an antagonistic fashion. (a, b) Light microscope images of GUS‐stained gynoecia of p*CYCP3;1:GUS* (a) and p*CYCP3;2:GUS* (b) in wild‐type Col‐0 and *spt‐12* mutant backgrounds with no treatment (a and b, left‐hand side) and with CK (50 μM BAP) treatment (a and b, the right‐hand side). Scale bars represent 200 μm (full‐size gynoecia) and 100 μm (style magnifications). *n* = 3 biological replicates and ~25–30 gynoecia were analysed for each genotype and treatment. (c) Confocal images of tobacco leaf cells co‐infiltrated with either *pCYCP3;1:CYCP3;1‐Cit* or *pCYCP3;2:CYCP3;2‐Cit* plus *35S::SPT‐RFP* or *35S::NLS‐RFP* as depicted on the panels. GFP, RFP, Brightfield and merged images are shown. *n* = 3 biological replicates. Note, *CYCP3s* are expressed only in the absence of SPT. (d) Confocal images of tobacco leaf cells co‐infiltrated with *pCYCP3;1* ^*G‐mut*^:*CYCP3;1:Cit* and *35S::SPT‐RFP* (top panels); *35S::SPT‐RFP* co‐expressed with either single mutated versions of *pCYCP3;2* (*pCYCP3;2* ^*I G‐boxMut*^:*CYCP3;2‐Cit* and *pCYCP3;2* ^*II G‐boxMut*^:*CYCP3;2‐Cit*) (middle panels) or double mutated version (*pCYCP3;2* ^*I+II G‐boxMut*^:*CYCP3;2‐Cit*) (bottom panels). GFP, RFP, Brightfield and merged images are shown. *n* = 3 biological replicates.

We constructed and analysed *CYCP3s* transcriptional GUS‐fusion (*pCYCP3;1:GUS* and *pCYCP3;2:GUS*) in WT and *spt‐12* backgrounds. In line with the hypothesised repressive activity mediated by SPT, neither *CYCP3s* were expressed in the wild‐type style (Figure 2a,b). Interestingly, *CYCP3;1* was expressed in the adaxial tissue of the bilateral ovary, the endocarp‐*a*, from stage 9 of gynoecium development onwards (Figure 2a; Figure S2a). *CYCP3;2* was not expressed in any tissue of the developing gynoecium (Figure 2b) until much later in development, after the patterning of the style is completed (Figure S2b). Optical microscopy analysis revealed that both *CYCP3s* were upregulated in the *spt* mutant background, predominantly at the apex of the developing organ, where the unfused carpels break radial symmetry of the style (Figure 2a,b).

In addition, we performed co‐expression experiments in tobacco leaves for both CYCP3s genomic sequence driven by their native promoters and fused to CITRINE (Cit) (*pCYCP3;1:CYCP3;1‐Cit* and *pCYCP3;2:CYCP3;2‐Cit*), co‐expressed with either *35S::SPT‐RFP* or *35::NLS‐RFP* (Figure 2c). We observed that CYCP3s were expressed in tobacco epidermal cells and localised in both the nucleus and cytoplasm, only when co‐expressed with the *35::NLS‐RFP* construct. By contrast, the co‐expression of *35S::SPT‐RFP* with either *pCYCP3s:CYCP3s:Cit* caused the elimination of CYCP3s expression (Figure 2c). To prove the downregulation mediated by SPT was operating at the transcriptional level, we mutated the single and double G‐box elements included in the *CYCP3;1* and *CYCP3;2* promoters, respectively (Figures 1g and 2d). Mutation of the single G‐box element in the *CYCP3;1* promoter (*pCYCP3;1* ^*G‐mut*^:*CYCP3;1:Cit*) co‐expressed with *35S::SPT‐RFP* rescued the expression of CYCP3;1 (Figure 2d). For CYCP3;2, mutation of each single G‐box element (*pCYCP3;2* ^*I G‐boxMut*^:*CYCP3;2:Cit* and *pCYCP3;2* ^*II G‐boxMut*^:*CYCP3;2:Cit*) was not sufficient to do so, while the simultaneous mutation in both G‐boxes (*pCYCP3;2* ^*I+II G‐boxMut*^:*CYCP3;2:Cit*) enabled the recovery of CYCP3;2 when co‐expressed with *35S::SPT‐RFP* in tobacco leaves (Figure 2d).

Furthermore, SPT interacts genetically and physically with another bHLH transcription factor INDEHISCENT (IND), which binds to the E‐Box variant (CACGCG) (Robinson et al., 2000) (Figure 1g). According to the synergistic activity of SPT and IND, *CYCP3s* expression was found to be upregulated in *ind‐2* apical style *in vivo* (Figure S2c) and downregulated *in vitro* in seedlings of the *35S::IND:GR* overexpression line followed by 3 and 24 h of IND induction by dexamethasone (DEX) treatments (Figure S2d).

Altogether these data demonstrate that SPT and IND both downregulate *CYCP3;1* and *CYCP3;2* expression.

In line with several CK‐binding sites (GARP AGATT(T/C)) (Xie et al., 2018) present in the promoters of *CYCP3s* (Figure 1g), next we tested the role of CKs on the *in vivo* expression of *CYCP3s* at the style region. CK treatments (6‐BAP) followed by GUS staining of *pCYCP3;1:GUS* and *pCYCP3;2:GUS* reporters revealed that both *CYCP3s* were ectopically upregulated, specifically at the gynoecium apex, following exogenous hormonal applications (Figure 2a,b), mimicking the expression seen in the *ind* and *spt* single backgrounds without CK treatments. The transcriptional upregulation of *CYCP3;1* transcript by CK was also confirmed by quantitative reverse transcription‐polymerase chain reaction (qRT‐PCR) experiments performed on WT inflorescences treated with mock and BAP (Figure S2e), while the levels of *CYCP3;2* on the other side remained unchanged, possibly due to differential regulation in other floral organs or tissues. Furthermore, *CYCP3s*' ectopic upregulation in the style region was further enhanced in the *spt* background *in vivo* by CK treatments, supporting an antagonistic and additive effect of SPT and CK on the control of *CYCP3s*' expression (Reyes‐Olalde, Zuniga‐Mayo, Serwatowska, et al., 2017; Schuster et al., 2015) (Figure 2a,b).

To further test the CK‐mediated control of *CYCP3s* expression, we used an inducible constitutive active form of a B‐type Arabidopsis Response Regulators (ARR‐B), ARR1 (*35S::ARR1ΔDDDK:GR*) (Sakai et al., 2001, 2008), a positive signalling component of the CK pathway and compared DEX‐treated inflorescences of *35S::ARR1ΔDDDK:GR pCYCP3;2:GUS* and *35S::ARR1ΔDDDK:GR pCYCP3;1:GUS* to mock‐treated controls (Figure S2f,g). These experiments revealed that *CYCP3;2* expression was ectopically induced in the endocarp‐*a* (mimicking the expression of *CYCP3;1*) and in the style by the CK signalling, while CYCP3;1 was not affected by the constitutive activation of ARR1. This *in vivo* analysis showed that induction of ARR1 is sufficient to upregulate at least *CYCP3;2* expression in the gynoecium tissues (Figure S2g), while CYCP3;1 might be regulated by other type‐B ARRs.

Altogether, our data provide the first direct *in vivo* evidence for an opposite transcriptional regulation of two potential core players of the cell cycle by SPT and CK during organ development, in a coherent forward loop type II (Alon, 2007).

### Ectopic activity of CYCP3;1 and CYCP3;2 break radial symmetry at the gynoecium apex

**Figure 3:**
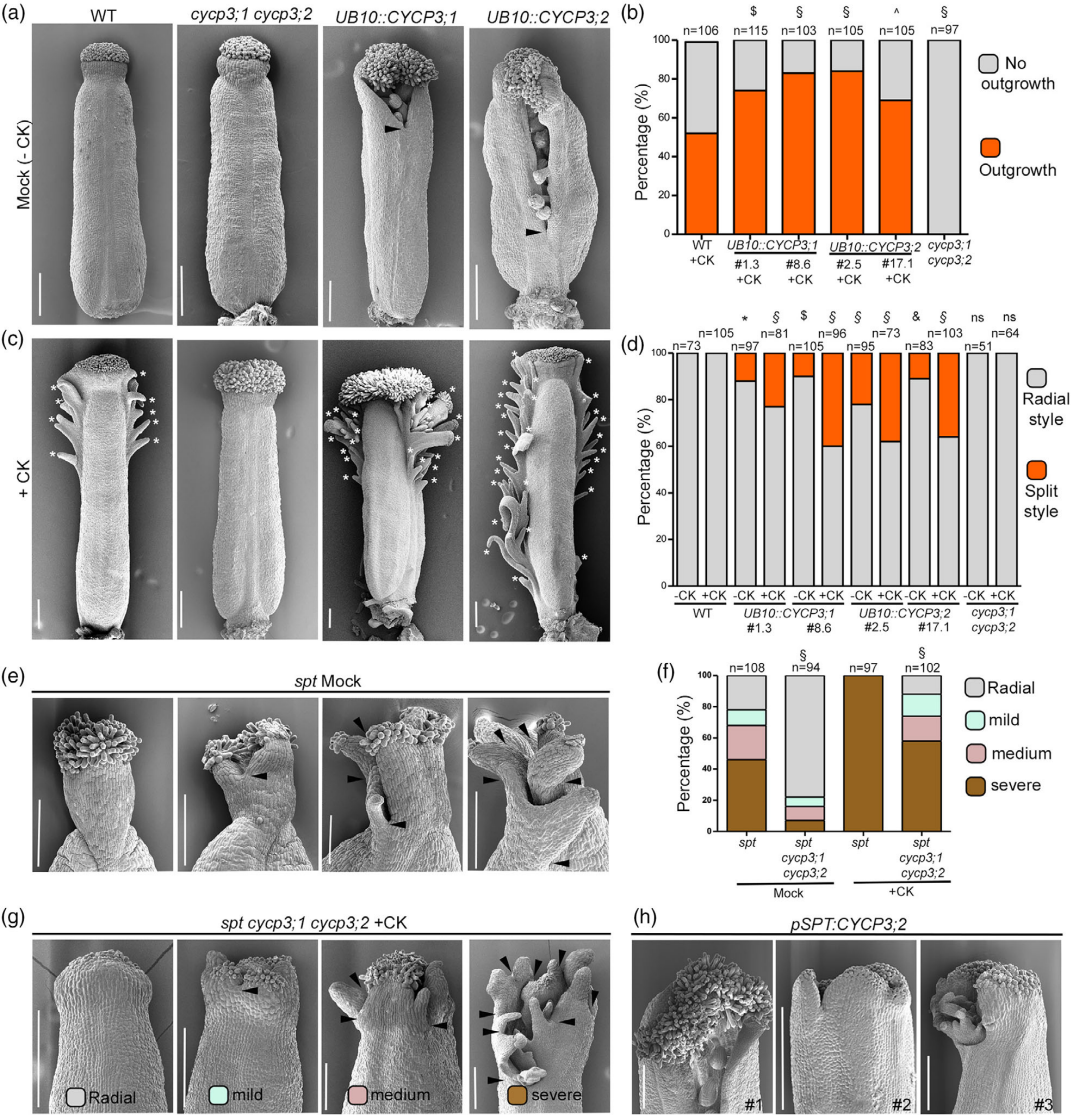
CYCP3s function is necessary and sufficient to control style radial symmetry. (a, c) Scanning electron microscope (SEM) images of mock (−CK) (a) and cytokinin (CK) (+CK) (c) treated gynoecia of wild‐type Col‐0, double *cycp3s* CRISPR mutant (*cycp3;1cycp3;2*) and a representative homozygous overexpressing lines of *UB10::CYCP3;1:Myc* (#1.3) and *UB10::CYCP3;2:Myc* (#2.5). Black arrowheads indicate the split‐style phenotype, while the white asterisks indicate ectopic growth. Scale bars are 200 μm. (b) Bar chart of quantification (percentage) of ectopic outgrowths phenotype in +CK treated gynoecia of wild‐type Col‐0, *cycp3;1cycp3;2* and two independent overexpressing homozygous lines of *UB10::CYCP3;1:Myc* (#1.3 and #8.6) and *UB10::CYCP3;2:Myc* (#2.5 and #17.1). Grey bars represent ‘no outgrowths’ and orange bars represent ‘outgrowths’. 2 × 2 contingency table followed by Fisher's exact Chi square test was used to compare phenotypic classes. Two‐tailed *P* values are as follows: Col‐0 CK versus #1.3 CK, *P* = 0.0032 ($), Col‐0 CK versus #8.6, Col‐0 CK versus #2.5 and Col‐0 CK versus *cycp3;1 cycp3;2*, *P* < 0.00001 (§) and Col‐0 CK versus #17.1, *P* = 0.04 (^). Three biological replicates have been performed; results of one representative biological replicate are plotted. Number of samples (*n*) analysed for each genotype/treatment are written on top of bars. (d) Bar chart of quantification (percentage) of radial (grey bars) and split (orange bars) phenotypes of −CK and +CK treated gynoecia of wild‐type Col‐0, *cycp3;1cycp3;2* and two independent overexpressing homozygous lines of *UB10::CYCP3;1:Myc* (#1.3 and #8.6) and *UB10::CYCP3;2:Myc* (#2.5 and #17.1). 2 × 2 contingency table followed by Fisher's exact Chi square test was used to compare phenotypic classes. Two‐tailed *P* values are as follow: Col‐0 mock versus #1.3 mock, *P* = 0.0003 (*); Col‐0 mock versus #8.6 mock, *P* < 0.006 ($); Col‐0 mock versus #2.5 mock, *P* < 0.00001 (§); Col‐0 mock versus #17.1, *P* < 0.0007 (&) Col‐0 mock versus *cycp3;1 cycp3;2* mock *P* = 1 (ns, non‐significant); Col‐0 Ck versus #1.3 CK, Col‐0 Ck versus #8.6 CK, Col‐0 Ck versus #2.5 CK, Col‐0 CK versus #17.1 CK, *P* < 0.00001 (§) and Col‐0 CK versus *cycp3;1 cycp3;2* CK, *P* = 1 (ns, non‐significant). Three biological replicates have been performed; results of one representative biological replicate are plotted. Number of samples (*n*) analysed for each genotype/treatment is written on top of bars. (e) SEM images of styles of *spt* mock (internal control) gynoecia showing four different categories of phenotypes, including radial (grey), mild split (blue), medium split (pink) and severe split (brown). Scale bars are 200 μm. (f) Bar chart showing percentage of radial and split (mild, medium, severe) phenotypes of mock and CK treated (+CK) *spt* (internal control) and *spt cycp3;1 cycp3;2* gynoecia. 2 × 2 contingency table followed by Fisher's exact Chi square test was used to compare phenotypic classes. Two‐tailed *P* values are as follow: *spt* mock versus *spt cycp3;1 cycp3;2* mock and *spt* +CK versus *spt cycp3;1 cycp3;2* +CK, *P* < 0.00001 (§). Three biological replicates have been performed; results of one representative biological replicate are plotted. Number of samples (*n*) analysed for each genotype/treatment is written on top of bars. (g) SEM images of styles of *spt cycp3;1 cycp3;2* gynoecia treated with CK (+CK) showing four different categories of phenotypes, including radial (grey), mild split (blue), medium split (pink) and severe split (brown). Scale bars are 200 μm. (h) SEM images of styles of gynoecia of 3 independent T1 lines of *pSPT:CYCP3;2:HA* (#1, #2, #3). Scale bars are 200 μm.

It has been previously shown that CYCP3s control cell proliferation by supporting the mitotic index of the root meristematic cells (Chen et al., 2020). In addition, in the gynoecium medial tissues, CK induces cell proliferation (Ramos‐Pulido & de Folter, 2025). To understand whether CYCP3s function mediates cell proliferation triggered by CK, we tested whether a double *cycp3s* loss‐of‐function mutant displays resistance to CK applications. We constructed a double CRISPR‐Cas9 mutant for both CYCP3s (Figure S3a–d) and treated it with exogenous CK applications. In this double *cycp3s* CRISPR mutant, guides were directed upstream of the cyclin‐box motif (necessary for Cyclins binding to CDKs) which is contained in the first exon of both CYCP3s (Figure S3a,b). The premature stop codon presumably produces truncated protein forms, which we predict to change CYCP3;1 from 220 amino acid (aa) residues into an allele we named *cycp3;1‐1* of 96 aa and CYCP3;2 from 230 aa to the allele *cycp3;2‐1* of 90 aa (Figure S3c,d). This double mutant showed a shorter primary root length and a reduction in the number of cortical cells in the root meristem (Figure S4a–c), in agreement with a previously characterised mutant with reduced levels of both *CYCP3s* transcript produced by RNAi (Chen et al., 2020). Overall morphology and tissue patterning of our *cycp3s* CRISPR double mutant plants and gynoecia remain intact (Figure 3a; Figure S4d). However, SEM analysis of wild‐type and *cycp3;1 cycp3;2* double mutant gynoecia treated with mock and CK showed the absence of ectopic growth from the medial tissues of the *cycp3s* CRISPR double mutant ovary and style (Figure 3a–c), suggesting that CK‐induced proliferation output in the gynoecium requires CYCP3s function.

**Figure 4:**
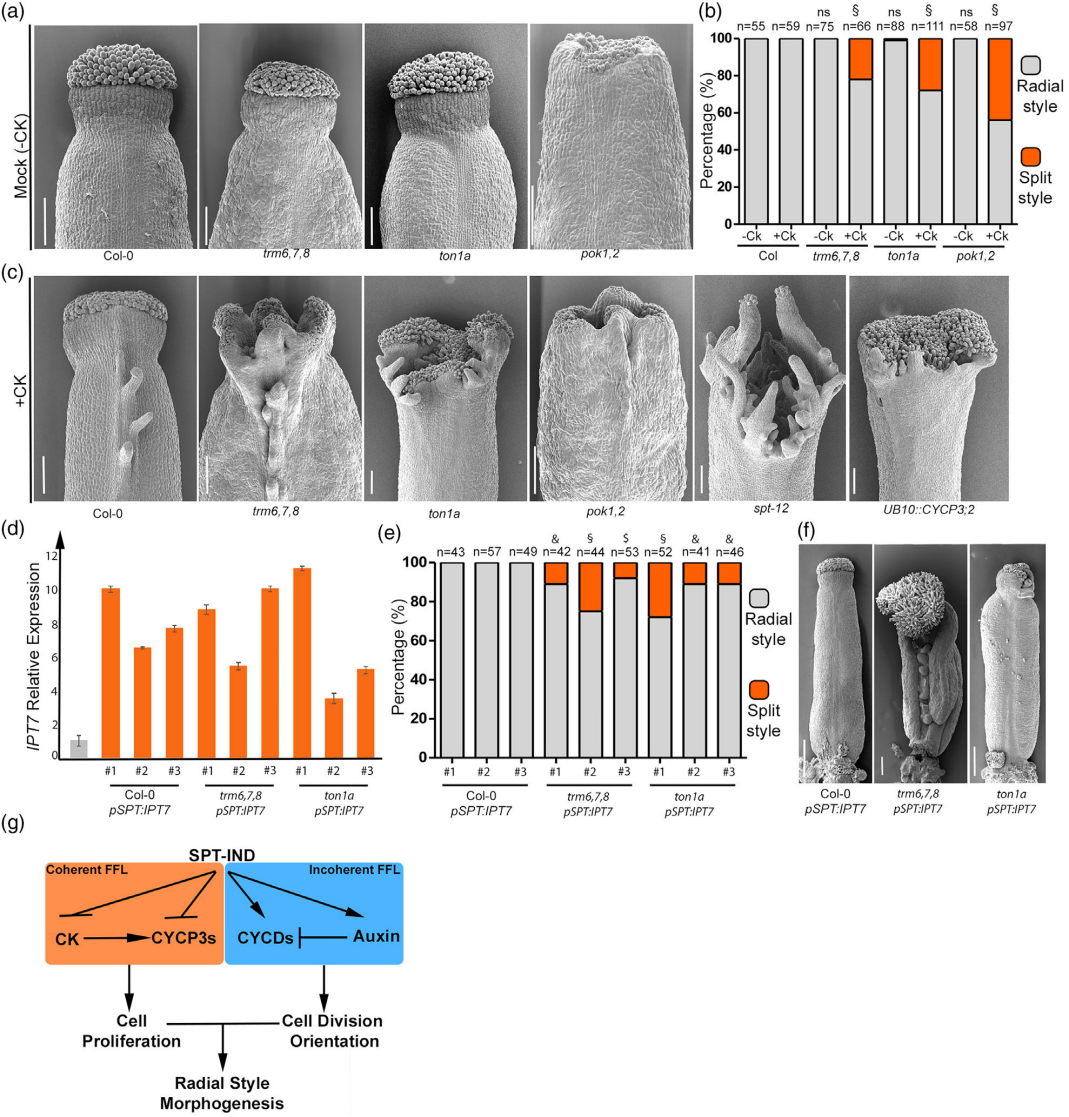
Cytokinin applications impact style morphology in cell division orientation mutants. (a) Scanning electron microscope (SEM) images of mock (−CK) treated gynoecia of wild‐type (Col‐0) and mutants in cell division orientation (*trm6,7,8, ton1a, pok1,2*) showing radial styles. Scale bars represent 100 μm. (b) Bar chart showing quantification (percentage) of radial (grey bars) and split (orange bars) style phenotypes of −CK and +CK treated gynoecia of Col‐0, *trm6,7,8, ton1a* and *pok1,2*. 2 × 2 contingency table followed by Fisher's exact Chi square test was used to compare phenotypic classes. Two‐tailed *P* values are as follow: Col‐0 mock versus *trm6,7,8* mock, Col‐0 mock versus *ton1a* mock and Col‐0 mock versus *pok1,2* mock, *P* = 1 (ns, non‐significant); Col‐0 cytokinin (CK) versus *trm6,7,8* CK, Col‐0 CK versus *ton1a* CK, Col‐0 CK versus *pok1,2* CK, *P* < 0.0001 (§). Three biological replicates have been performed; results of one representative biological replicate are plotted. Number of samples (*n*) analysed for each genotype/treatment is written on top of bars. (c) SEM images of CK treated (+CK) Col‐0, *trm6,7,8, ton1a, pok1,2, spt‐12* and *UB10::CYCP3;2* gynoecia. Scale bars are 100 μm. (d) Bar chart of qRT‐PCR showing relative expression levels of CK biosynthesis gene *IPT7* in 3 independent T1 lines of *pSPT:IPT7* in Col‐0, *trm6,7,8* and *ton1a* backgrounds. Expression levels were normalised against *UBIQUITIN10*. The experiment was performed once on three independent transgenic lines per construct, with four technical repeats. Values shown are means ± SEM. (e) Bar chart showing quantification (percentage) of radial (grey bars) and split (orange bars) style phenotypes of Col‐0, *trm6,7,8* and *ton1a* gynoecia transformed with *pSPT:IPT7* construct. Three independent T1 lines for each background are plotted. Number of gynoecia analysed for each genotype are shown on top of respective bars. 2 × 2 contingency table followed by Fisher's exact Chi square test was used to compare phenotypic classes. Two‐tailed *P* values are as follow: Col‐0 *pSPT:IPT7* versus *trm6,7,8 pSPT:IPT7* line *#1* and Col‐0 *pSPT:IPT7 versus ton1a* lines #2 and #3, *P* < 0.0007 (&); Col‐0 *pSPT:IPT7* versus *trm6,7,8 pSPT:IPT7* line *#2* and Col‐0 *pSPT:IPT7* versus *ton1a pSPT:IPT7* line *#1, P* < 0.00001 (§), Col‐0 *pSPT:IPT7* versus *trm6,7,8 pSPT:IPT7* line #3, *P* < 0.006 ($). (f) SEM images of representative gynoecia from one independent transgenic T1 line of Col‐0, *ton1a* and *trm6,7,8* transformed with *pSPT:IPT7* construct. Scale bars are 200 μm. (g) Schematic model showing the regulatory network of *CYCP3s* and *CYCDs* expression by bHLH TFs (SPT‐IND) and hormones CK and auxin forming a coherent and incoherent feed‐forward loop, respectively, to regulate radial style morphogenesis.

To test whether CYCP3s function is sufficient to break radial symmetry at the style, we overexpressed CYCP3;1 and CYCP3;2 (*UB10::CYCP3;1:Myc* and *UB10::CYCP3;2:Myc*) (Figure S3e,f). While there was no ectopic effect of CYCP3s overexpression on plant architecture (Figure S4e), a close analysis of their gynoecia by SEM revealed that a population of *CYCP3;1* and *CYCP3;2* overexpressing lines showed a consistent split‐style phenotype (Figure 3a,d). In line with a positive role in CK signalling, CK applications increased the frequency of the split style observed in the *UB10::CYCP3s:Myc* lines to 44%, while it had no effect in the wild‐type background (Figures 3a,d and 4c) as well as the ectopic outgrowths arising from the ovary (Figure 3b,c). These results are in line not only with the ectopic expression of *CYCP3s* in the style region following CK applications (Figure 2a,b), but also with stereo microscope analysis of GUS‐stained gynoecia of *pCYCP3s:GUS* showing ectopic expression of *CYCP3s* in the medial outgrowths arising from the ovary (Figure S2h) following prolonged CK treatments. These results further corroborate a functional role for CYCP3s in controlling cell proliferation and response to CK during gynoecium development, in particular at the style region for the control of style morphology.

Next, we asked whether the ectopic *CYCP3s* expression observed in the *spt* split‐style was causative of its bilateral phenotype and hypersensitivity of the *spt* style phenotype to CK applications. We analysed *spt cycp3;1 cycp3;2* triple mutant gynoecia by SEM and quantified the presence of bilateral versus radial style compared to a segregating *spt* control. The analysis showed that proper fusion of the style displaying radial symmetry was largely restored in the *spt cycp3;1 cycp3;2* mutant gynoecia (78% radial and 22% bilateral styles) compared to the *spt* segregating control (22% radial and 78% bilateral styles) (Figure 3e,f). Furthermore, CK application experiments showed that the gynoecia apices of the triple *spt cycp3;1 cycp3;2* were partially resistant to hormonal treatments compared to the *spt* single mutant. In these experiments we divided the split‐style phenotype into three further categories to account for the severity and complexity of the cleft, as severe, medium and mild (Figure 3e–g). As previously shown, the *spt* style phenotype increased drastically with CK applications, leading to 100% of samples showing a severe split style compared to mock‐treated *spt* mutants (Figures 3f and 4c).

Mock‐treated *spt cycp3;1 cycp3;2* gynoecia showed a vast increase in radial, fused style (78%) and a reduced frequency of severe (7%), medium (9%) and mild (6%) split‐style phenotypes (Figure 3f). Although CK treatments still raised the frequency of severe (58%), medium (16%) and mild (14%) split styles, 12% of radial styles were still present after the treatment of *spt cycp3;1 cycp3;2* gynoecia, which is never the case for the *spt* single mutant (Figure 3f,g). These data demonstrate that CYCP3s promote ectopic cell proliferation in the *spt* mutant background via promoting the CK‐mediated cell division input and style morphology.

Lastly, to test whether ectopic CYCP3s function could break style radial symmetry by working cell‐autonomously in the SPT‐expression domain, we analysed *pSPT:CYCP3;2:HA* transgenic lines that showed overexpression of CYCP3;2 transcript (Figure S3g) and observed a split‐style phenotype that resembles that of both *spt* and *UB10::CYCP3s* mutants (Figure 3h). This corroborates a model where SPT represses *CYCP3;2* (and *CYCP3;1*) expression cell‐autonomously to fuse the apical carpels.

Altogether, our data show that the activity of CYCP3s is sufficient to break radial symmetry at the gynoecium apex and necessary to confer sensitivity to CK when SPT function is missing.

### Increasing CK levels affect symmetry establishment in mutants impaired in cell division orientation

SPT promotes the accumulation of auxin maxima foci, which in turn are important to maintain division orientation in the periclinal direction (Tasker‐Brown et al., 2024) to form a radial style (Carabelli et al., 2021; Moubayidin & Ostergaard, 2014). Division plane orientation in plants is established before mitosis and must be maintained throughout mitosis and cytokinesis (Facette et al., 2019). This process is often thought to occur when the PPB forms in the G2 phase of the cell cycle (Facette et al., 2019; Schaefer et al., 2017; Spinner et al., 1863) although the current scenario assigns the PPB a marginal role in determining cell division orientation, compared to the past view: the PPB would add robustness to the selection of the right division angle, rather than being a key determinant of cell division orientation *per se* (Schaefer et al., 2017).

Key components of the PPB establishment machinery such as TONNEAU 1A (TON1A) (Azimzadeh et al., 2008), the TON1 Recruiting Motif 6,7,8 (TRM6,7,8) (Schaefer et al., 2017) proteins and PPB maintenance PHRAGMOPLAST ORIENTING KINESIN 1 (POK1) and POK2 (Müller et al., 2006) were found among the presumptive SPT downstream targets (Table S1). Notably, although the PPB still forms in the *spt* background, its orientation is misplaced at the mutant developing style (Tasker‐Brown et al., 2024).

How cell division orientation governs carpels' apical fusion and style shape is unknown. To understand whether the correct orientation of cell division is required for style radialisation, we tested whether mutants impaired in cell division orientation would display a split‐style phenotype, similar to *spt*. To test the importance of cell division orientation in style development, we analysed by scanning electron microscopy (SEM) gynoecia of mutants in key components of the microtubule (MT)‐dependent mitotic structures important for the cell cycle: the PPB establishment, guided by TON1a and TRMs 6,7,8 (*ton1a‐1* and *trm6, trm7, trm8*) (Azimzadeh et al., 2008; Schaefer et al., 2017), and PPB maintenance guided by POK1 and POK2 (*pok1,2*) (Müller et al., 2006). None of the aforementioned mutants showed significant defects in style development (Figure 4a,b). This suggests that either cell division orientation is not essential *per se* for radial style morphogenesis, or a synergistic layer of control adds robustness to keep the orientation of newly forming cell walls perpendicular to the direction of growth (Tasker‐Brown et al., 2024), which ultimately guarantees apical‐basal anisotropic growth (Eldridge et al., 2016).

Because SPT promotes auxin accumulation and cell division orientation (Carabelli et al., 2021; Moubayidin & Ostergaard, 2014; Tasker‐Brown et al., 2024) and represses CK signalling (Schuster et al., 2015), dampening the mitotic potential at the marginal tissue (Reyes‐Olalde, Zuniga‐Mayo, Serwatowska, et al., 2017), we asked whether augmenting CK levels by exogenous applications in cell division orientation mutants would break radial symmetry and mimic the *spt* split style. SEM analysis of *ton1a*, *trm6,7,8* gynoecia of inflorescences treated with CK and mock, showed that CK applications uncovered a never‐seen‐before split phenotype of these mutants, leading to a significant percentage of split styles recovered in *ton1a*, *trm6,7,8* and *pok1,2* (Figure 4a–c). Moreover, these severe split‐style phenotypes displayed augmented complexity of their distal gynoecia apices, similar to *spt* gynoecia treated with CK (Figure 4c). In addition, tissue‐specific expression of the CK biosynthetic gene *IPT7* (Dello Ioio et al., 2007) driven by the SPT promoter (*pSPT:IPT7*) (Figure 4d) showed increased frequency of split‐style phenotypes observed in *ton1a* and *trm6,7,8* mutant backgrounds but not in the wild‐type control (Figure 4e,f), yielding similar results to those obtained with CK exogenous applications (Figure 4a–c).

Altogether, our results demonstrate a coordination between cell proliferation and division orientation to fuse the apical style.

In conclusion, our data supports a model where a key regulator of style development, SPT, provides robustness to the placement of the cell division angle by a local fine‐tuning of cell cycle progression and proliferation mediated via the control of two families of cyclins: CYCD1;1 and CYCD3;3 via auxin (Tasker‐Brown et al., 2024) and CYCP3;1 and CYCP3,2 via CK (Figure 4g), ultimately important for shaping radial symmetry at the style region.

## DISCUSSION

Altogether, our results demonstrate a role for the presumptive cell cycle regulators CYCP3;1 and CYCP3;2 in controlling radial symmetry establishment at the gynoecium apex, a critical step required for the final fusion of the two carpels in Arabidopsis reproductive development.

The bHLH transcription factor SPT has long been implicated in the antagonistic crosstalk between auxin and CK, yet the downstream activities of this network remain unclear. In particular, the molecular link between SPT and CK in regulating apical style fusion was previously unknown. Here, we demonstrate that the antagonistic roles of SPT and CK converge on the regulation of *CYCP3s* gene expression in an opposing manner: SPT directly represses *CYCP3*s expression in the style region, while CK promotes their expression, forming a coherent FFL type II (Alon, 2007). This antagonistic regulation of *CYCP3s* by SPT and CK aligns with previous findings that CYCP3s are positive targets of CK signalling in the shoot apical meristem (Xie et al., 2018) and are negatively regulated by the SPT‐interacting factor, IND (Simonini et al., 2016). While SPT modulates various cellular processes, our results clarify one mechanism underlying the hypersensitivity of *spt* mutants to CK treatments, which vastly exacerbates the apical infusion of the carpels. Specifically, our results indicate that repression of *CYCP3s* expression by SPT in the style region is one of the pathways controlled by this master regulator, ensuring proper style morphology. Accordingly, the overexpression of *CYCP3s* displays a (proportion of) phenotype resembling that of *spt* mutants, namely a split style (Figure 3a,d). Nevertheless, not all CYCP3s‐overexpressing gynoecia exhibit unfused styles, suggesting that the cellular activity regulated by CYCP3s represents only one of multiple functions required for proper radial morphology of the style.

Notably, our comprehensive list of putative SPT targets does not include ARR1 but does comprise the closely related B‐type ARR, ARR10 (Table S1). This finding raises the intriguing possibility that SPT may operate within context‐specific transcriptional regulatory networks, enabling its dual role in CK signalling across distinct gynoecium tissues: repressing CK output in the style to facilitate carpel apical fusion, while enhancing CK signalling in the medial ovary tissues to support the development of the transmitting tract and the carpel margin meristem (Reyes‐Olalde, Zuniga‐Mayo, Marsch‐Martinez, & de Folter, 2017; Reyes‐Olalde, Zuniga‐Mayo, Serwatowska, et al., 2017).

Coherent FFLs have been suggested to add robustness to signalling networks by making downstream nodes more resistant to perturbations compared to single incoherent FFLs (Le & Kwon, 2013). Thanks to the coherent FFL type II identified in this work, the induction of *CYCP3* genes by CK is attenuated in the presence of functional SPT. In this loop, *CYCP3s* expression is buffered because SPT represses *CYCP3s* both directly and indirectly, by also repressing CK, which is itself a positive regulator of CYCP3s. As a result, even when CK levels rise, SPT mitigates the effect by counteracting CK‐driven *CYCP3s* activation. This dual repression ensures that *CYCP3s* expression remains controlled, thereby safeguarding proper style development.

We previously showed that SPT (and IND) also participate in an incoherent FFL with auxin to regulate the expression of D‐type cyclins, potentially coordinating G1 cell cycle progression and the orientation of cell division (Tasker‐Brown et al., 2024). Interestingly, while incoherent FFLs accelerate the response of target genes, coherent FFLs tend to delay them (Mangan & Alon, 2003). This suggests a model where SPT finetunes cell division activities by interacting with auxin and CK signalling through these two types of FFLs (Figure 4g).

Carpel fusion is essential for efficient fertilisation and seed production. Growth analyses of the wild‐type gynoecium reveal that strong anisotropic growth in the apical‐basal direction drives the development of epidermal tissue in the style region (Eldridge et al., 2016; Gomez‐Felipe et al., 2024). Cell clones in the style expand longitudinally and divide in the transverse anticlinal direction (Eldridge et al., 2016). Our recent analysis of the *spt* mutant bilateral style revealed abnormalities in cell division orientation control at the onset of the cleft, where an auxin maximum accumulates to drive carpel fusion (Tasker‐Brown et al., 2024). To test this model, we investigated whether mutants with aberrant cell division orientation, such as *ton1a* and *trm6,7,8*, displayed defects in style morphology. Our genetic analysis showed that defects in cell division orientation alone are insufficient to disrupt radial symmetry in the style, suggesting an additional layer of control that enhances robustness. We found that increasing CK levels in PPB‐defective mutants, via exogenous CK application or endogenous CK production by the *IPT7* gene, resulted in severe style defects similar to the *spt* phenotype. This confirms that CK impacts radial style formation in cell division mutant backgrounds most probably by influencing cell proliferation. It also highlights a link between CK action and key players in PPB establishment, such as TON1a (Azimzadeh et al., 2008) and TRM6,7,8 (Schaefer et al., 2017), which add robustness to cell division plane placement during style radial patterning. This may reflect a specific role for CK in carpel fusion control, where proper cell division orientation requires additional regulation to ensure successful carpel fusion and reproductive fitness.

We demonstrated that CYCP3s function might promote CK‐mediated cell proliferation, as CRISPR mutants with *cycp3;1‐1 cycp3;2‐1* loss‐of‐function and CYCP3s‐overexpressing lines were resistant and hypersensitive to CK applications, respectively, as measured by the extent of outgrowths in the ovary region (Figure 3b). However, further experiments are required to elucidate how CYCP3s control cell proliferation in the gynoecium. The role of CYCP3s in the cell cycle also remains unclear. It has been previously suggested that CYCP3;1 may function during the G2 phase of the cell cycle, as it interacts with and activates CDKB2;1, a cyclin‐dependent kinase specifically expressed during the G2 and M phases (Chen et al., 2020). If CYCP3s act downstream of CK signalling, it is plausible to speculate that their function occurs during the G2 phase, a critical temporal window in which CK promotes cell proliferation in the shoot apical meristem (Yang et al., 2021). This scenario would also be compatible with a potential, direct or indirect, role for CYCP3s in PPB formation, which also takes place during the G2 phase (Facette et al., 2019; Spinner et al., 1863).

In addition to their presumptive proliferation role, CYCP3s may also affect other processes, such as controlling the orientation of cortical or cytoplasmic MTs. Recent research has shown that a radial arrangement of cytoplasmic MTs precedes PPB formation and enables cells to sense their geometry, facilitating symmetric division and the precise partitioning of cell volume into two daughter cells (Melogno et al., 2024).

Since increasing cell proliferation in cell division orientation mutants is sufficient to disrupt radial symmetry in the style, this suggests that CK promotes growth and/or cell expansion in the medio‐lateral direction, antagonising the auxin‐mediated apical‐basal style expansion. This may shift growth from anisotropic to isotropic, breaking the standard rules of growth and division at the gynoecium apex (Eldridge et al., 2016; Gomez‐Felipe et al., 2024). Supporting this, CK has been shown to regulate directional cell expansion/growth via cortical MT rearrangement (Montesinos et al., 2020).

A dual role for CYCPs is consistent with the characterisation of CYCP homologs in the unicellular species *Trypanosoma brucei* (CYC2, CYC4, CYC5 and CYC7). When these cyclins are silenced, cells arrest in the G1 phase, and incorrect MT assembly leads to posterior axis bifurcation (Liu et al., 2013).

Taken together with our previous findings (Tasker‐Brown et al., 2024), this work shows that SPT directly finetunes the expression of CYCP3s in an opposite manner to CK and integrates fundamental cellular processes, including cell division orientation, G1 phase progression and cell proliferation, to orchestrate the morphogenesis of a radial style (Figure 4g).

## MATERIALS AND METHODS

### Plant materials and growth conditions

The following mutants in the wild‐type ecotype Columbia (Col‐0) were used in the study: *spt‐12* (Heisler et al., 2001), *ind‐2* (Liljegren et al., 2004), *trm6,7,8* (Schaefer et al., 2017), *ton1a* (Azimzadeh et al., 2008), *pok1,2* (Müller et al., 2006), *35S::ARR1ΔDDK:GR* (Sakai et al., 2001) and *35S::IND:GR* (Sorefan et al., 2009). *spt‐12*, *ind‐2* and *35S::ARR1ΔDDK:GR* mutants were crossed with transcriptional fusion lines *pCYCP3;1:GUS* and *pCYCP3;2:GUS*. Homozygous *cycp3;2*‐1 mutant was crossed to *spt‐12* mutant and the resulting double mutant was crossed to homozygous *cycp3;1‐1* mutant to obtain a *spt cycp3;1 cycp3;2* triple mutant as well as a segregating *cycp3;1 cycp3;2* double mutant. The SPT complementation line *spt‐12/SPT*::*SPT*–*sYFP* line used for ChIP‐Seq analysis in this study was described previously (Jiang et al., 2024). Plants were grown in JOHN INNES F2 STARTER soil mix (100% Peat, 4 kg m^−3^ dolomitic limestone, 1.2 kg m^−3^ osmocote start) in a controlled environment room (CER) set at 22°C, with 80% relative humidity, in long day conditions (16 h light/8 h dark).

### Gynoecium and seedling treatments

To test the phenotypical effect of CK on gynoecia, treatments of Col‐0, *UB10::CYCP3;1:Myc, UB10::CYCP3;2:Myc, cycp3;1 cycp3;2, spt‐12, spt cycp3;1 cycp3;2, trm6,7,8, ton1a* and *pok1;2* plants were performed by spraying with 50 μM 6‐Benzylaminopurine (BAP) (Merck, Darmstadt, Hesse, Germany) and mock (NaOH), twice a week (four times in total). The first spray was done 1 week after bolting, and samples were collected after 4 days of the last spray. To test the phenotypical effect of naphthylphthalamic acid (NPA) treatment on Col‐0 style, plants were treated with 100 μM NPA (Duchefa Biochemie N0926) and mock (Absolute ethanol, Merck), twice a day (morning and evening); samples were collected after 1 week of treatment. For expression analysis via GUS staining, homozygous plants of *pCYCP3;1:GUS*, *pCYCP3;2:GUS, spt‐12 pCYCP3;1:GUS, spt‐12 pCYCP3;2:GUS, ind‐2 pCYCP3;1:GUS* and *ind‐2 pCYCP3;2:GUS*, were sprayed with 50 μM BAP for two consecutive mornings and samples for RNA extractions were collected the third morning. For analysing the expression of *pCYCP3;1:GUS* and *pCYCP3;2:GUS* in ectopic outgrowths from the ovary, homozygous plants of *pCYCP3;1:GUS* and *pCYCP3;2:GUS* were sprayed with 50 μM BAP (Merck) and mock (NaOH), twice a week (four times in total) as described above. For Dex treatments of *35S::ARR1ΔDDK::GR pCYCP3;1:GUS* (F1) and *35S::ARR1ΔDDK::GR pCYCP3;2:GUS* (F1) for GUS staining analysis, plants were sprayed with 10 μM DEX (Merck, #218928) for two consecutive mornings, followed by sample collection on the third morning. Silwet L‐77 (0.015%) was used in all spray treatments. For DEX treatment of the *35S::IND:GR* line for qRT‐PCR analysis, seedlings were grown vertically on Murashige and Skoog (MS) media for 5 days before shifting to DEX (10 μM) or Mock (DMSO, Merck) containing MS plates. Samples for RNA extractions were collected after 3 and 24 h of mock and DEX treatments.

### DNA constructs

#### UB10‐driven overexpressing CYCP3s lines

Constructs of *UB10::CYCP3;1:Myc* and *UB10::CYCP3;2:Myc* were assembled using Golden Gate modular cloning method (Engler et al., 2014) as follows: The genomic coding sequence (CDS) of CYCP3;1 (777 bp) and CYCP3;2 (816 bp) were amplified from genomic Col‐0 DNA using the primers pairs CYCP3;1_gORF_F/CYCP3;1_gORF_R and CYCP3;1_gORF_F/CYCP3;1_gORF_R, respectively (no stop codon was included in the reverse primers). A proofreading Taq (Q5, New England Biolabs, NEB, Ipswich, MA, USA) was used for the PCR reaction. The amplification products were run on a 0.8% agarose gel to assess their size. The rest of the PCR reaction was purified using the (QIAquick PCR and Gel cleanup kit, QIAGEN, Venlo, Netherlands) and used in combination with the L0 Golden Gate vector pICSL01005 using Bbs1 (Merck) and T4 ligase (Merck). Constructs were transformed to *Escherichia coli* (DH5α) competent cells and positive colonies were selected on Spectinomycin (Spec^R^) (Merck) LB plates. Plasmid DNA was extracted using NucleoSpin® Plasmid kit by MACHEREY‐NAGEL, enzymatic digestions were done using BsaI (NEB) and absence of mutations was confirmed by Eurofins sequencing (OVERNIGHT Mix2Seq kit). To generate level 1 constructs: amplicons of Level 0 modules, including the *UB10* promoter (pICSL12015), Level 0 vectors of pICSL01005_*CYCP3;1* and pICSL01005_*CYCP3;2*, C‐terminal *Myc* tag (pICSL50010) and *NOS* terminator (pICH41421) were combinatorially assembled into the Level 1 acceptor backbone (pICH47742) using a digestion and ligation (dig‐lig) protocol with the type II restriction enzyme *Bsa*I and T4 DNA ligase. Both constructs were transformed into *E. coli* (DH5α) strain. The transformed cells were selected on LB medium (Carb^R^) and incubated overnight at 37°C. Cultured colonies were screened by mini‐prep followed by restriction digestion using *Xba*I and *Hind*III enzymes. Selected colonies for each construct were confirmed by Eurofins sequencing. The Level 1 modules, *UB10::gORFcycp3;1:4xMyc:NOS* and *UB10::gORFcycp3;2:4xMyc:NOS*, in‐planta *HYG* resistance cassette (pICSL11059) and linker Ele2 (pICH41744) were assembled into the Level 2 acceptor backbone (pICSL4723) by the ‘dig‐lig protocol’ for the final Level 2 assembly using *Bbs*I (*Bpi*I) and T4 DNA ligase enzymes. Incubation of the reaction in a thermocycler (PCR), bacterial transformation, screening of colonies (Kan^R^ LB plates) followed by restriction digestion (using *Hind*III & *Pst*I) and confirmation of colonies by Eurofins sequencing was done as aforementioned. Col‐0 plants were transformed with each of the two constructs using the flower dip method (Clough & Bent, 1998) and *Agrobacterium tumefaciens* strain *GV3101*. Positive transformants were selected on Hyg. In T1, Hyg‐resistant plants segregating 1:3 were selected (one copy of the construct). In T2, homozygous Hyg‐resistant plants were selected and used for further analysis.

#### SPT‐driven overexpression of CYCP3;2 and IPT7 lines

*pSPT:gCYCP3;2:HA* construct was generated by In‐Fusion cloning as follows: L0 gORF of *CYCP3;2* as constructed above for *UB10::CYCP3;2:Myc* construct was digested with a primer pair CYCP3;2‐F (KpnI)/CYCP3;2‐R (XhoI) (see Table S2) and confirmed by Eurofins sequencing. CYCP3;2 was then cloned into pre‐digested linearised *pSPT–pCambia1305‐3xHA* (4980 bp) vector (gifted by Yuxiang Jiang from host lab) (Jiang et al., 2024) by In‐Fusion ligation reaction using 5× In‐Fusion^®^ HD enzyme, thus generating *pSPT:CYCP3;2:HA* construct. All constructs were confirmed by sequencing and transformed into *A. tumefaciens* strain *GV3101* for plant transformation in Col‐0 background. Positive T1 lines were selected on Basta^R^ MS plates and used for further analysis.

*pSPT:IPT7* construct (for transformation into Col‐0, *trm6,7,8* and *ton1a*) was constructed similarly. Complete CDS of *IPT7* gene (990 bp) including the stop codon was amplified from Col‐0 CDS DNA using gene‐specific primers (see Table S2). *IPT7* CDS sequence was digested with primer pair IPT7‐F (KpnI)/IPT7‐R (XhoI) and cloned into pre‐digested linearised *pSPT–pCambia1305‐3xHA* (4980 bp) vector by In‐Fusion ligation reaction using 5× In‐Fusion^®^ HD enzyme. The absence of mutations was verified by Eurofins sequencing and the construct was transformed into *A. tumefaciens* strain *GV3101* for plant transformation. Positive T1 lines were selected on Basta^R^ MS plates and used for further analysis.

#### *cycp3;1‐1* and *cycp3;2‐1* CRISPR mutants

Single CRISPR Cas9 mutants for CYCP3;1 (*cycp3;1‐1*) and CYCP3;2 (*cycp3;2‐1*) were obtained as follows: Two guides were used for each gene (guide3: CTAGGAACGAGAGAATCAGC and guide1: GTATACCAAAGCCGGTCCAT for CYCP3;1; guide9: TGACCATCCAGTCATACCTA and guide6: GTACACTAAAGCCGGTCCTT for CYCP3;2) and included in specific forward primers to be cloned using the Golden Gate cloning technology.

For each *sgRNA*, we selected only those with an on‐target efficiency score greater than or equal to 0.6, indicating a high predicted capacity to specifically cleave the target DNA sequence. To further minimise off‐target effects—unintended cuts in other regions of the genome—the *sgRNA* sequences were analysed using Basic Local Alignment Search Tool (BLAST) to ensure no significant similarity to non‐target regions.

Each forward primer was used in combination with a universal reverse primer for PCR amplification using Phusion Polymerase Taq (30 cycles at 56°C for 10 sec). Amplification products were separated on a 2.5% agarose gel and purified using a gel filtration kit (Merck). CYCP3;1 guide3 and CYCP3;2 guide9 were cloned into the pICH47751 vector, while CYCP3;1 guide1 and CYCP3;2 guide6 were cloned into the pICH47761 vector. All resulting constructs were transformed in *E. coli* competent cells (DH5α) and selected on LB medium (Carb^R^). After plasmid extraction using the NucleoSpin^®^ Plasmid kit from the positive colonies and sequencing by Eurofins, the two guides for each gene were combined in an L2 reaction using the pICSL4723 destination vector, alongside the pICSL11015 (FastRed selection in plants), the CAS9 BCJJ358 vector and the linker Ele4 pICH41780. The *E. coli* positive colonies were selected on Kan^R^ LB plates. L2 plasmids were used to transform Col‐0 plants using the *Agrobacterium* infiltration methods. Individual T1 FastRED positive seeds were selected using a stereo fluorescent microscope (Leica M205FA). Genomic DNA was extracted by individuals T1 and T2 adult plants using the isopropanol method. Each DNA was used as a template to amplify a region across the two guides: for *cycp3;1‐1*, CYCP3;1_CRISPRseq_F and CYCP3;1_CRISPRseq_R (see Table S2) were used to amplify a region of 851 bp, while for *cycp3;2‐1*, CYCP3;2_CRISPRseq_F and CYCP3;2_CRISPRseq_R (see Table S2) were used to amplify a region of 723 bp. Aliquots of the PCR products were run on a 1.8% agarose gel and the rest was cleaned up for sequencing using the respective CRISPRseq_F primers. Scrambled sequences at the guide positions onwards were considered edited and the corresponding plants' offsprings were grown to obtain a second generation of edited plants. In T2s, FastRED negative seeds were selected to eliminate the Cas9, the genomic DNA was extracted from adult plants and amplified as above. Sequencing results revealed homozygosis for a point mutation for *cycp3;1‐1* (line 6‐4) and a big deletion for *cycp3;2‐1* (line B2‐1) (see Figure S3).

Homozygote *cycp3;2*‐1 pollen was used to pollinate *spt‐12* mutant gynoecia to generate double mutant and the resulting screened double mutant's pollen was used to pollinate homozygote *cycp3;1‐1* mutant gynoecia to obtain a *spt cycp3;1 cycp3;2* triple mutant as well as a segregating *cycp3;1 cycp3;2* double mutant. Homozygosis for both CRISPR *CYCP3s* alleles was tested by genomic DNA extraction followed by sequencing, while the *spt‐12* wild‐type and T‐DNA alleles were screened by PCR using the primer pairs spt‐12 RP/spt‐12 LB for wild‐type and spt‐12 RP/spt‐12 LP for T‐DNA (see Table S2).

#### *pCYCP3;1:GUS* and *pCYCP3;2:GUS* transcriptional fusion lines

A fragment of 2949 bp upstream of the CYCP3;1 start codon was cloned to produce the GUS transcriptional fusion *pCYCP3;1:GUS* using the primer pair pCYCP3;1_FWD/pCYCP3;1_REV (see Table S2), while a fragment of 2845 bp upstream of the CYCP3;2 start codon was cloned to produce the GUS transcriptional fusion *pCYCP3;2:GUS* using the primer pair pCYCP3;2_F/pCYCP3;2_R (see Table S2). The PCR products were cloned into the destination L0 vector pICH41295 using the Golden Gate cloning strategy and then transformed into *E. coli* (DH5α). Positive colonies were selected on LB plates supplemented with Spec. Plasmid DNA was extracted using the NucleoSpin^®^ Plasmid kit, enzymatic digestions were performed to confirm the correct size of the inserts within the receiving plasmids and sequencing by Eurofins. L1 reactions were performed to combine each promoter to the GUS reporter by using the pICH47742 backbone, the pICH75111 vector containing the GUS sequence and the pICH41421 NOS terminator. The two resulting constructs were transformed in *E. coli* (DH5α) and the positive colonies screened on Carb^R^ LB plates. Enzymatic digestions and sequencing were performed to check the size and the correct junction of the GUS reporter to the promoters. L2 reactions were then performed to clone the *pCYCP3;1:GUS* and *pCYCP3;2:GUS* constructs in to the destination vector pAGM4273 alongside the vector pICSL11024 for the resistance cassette (Kan^R^) for selection in plants. The resulting vectors were transformed into *A. tumefaciens* strain *GV3101* before transformation into Col‐0 plants. T1 with single insertion and homozygote T2 transgenic lines were selected using resistance to KAN. Homozygous T2 lines were then crossed to the *spt‐12* and *ind‐2* mutants to obtain double homozygote lines (*spt‐12 pCYCP3;1:GUS; ind‐2 pCYCP3;1:GUS; spt‐12 pCYCP3;2:GUS*; and *ind‐2 pCYCP3;2:GUS*). Also *pCYCP3;1:GUS* and *pCYCP3;2:GUS* were crossed to *35S::ARR1ΔDDK:GR* to generate several F1s to be used for further analysis.

#### Cloning strategy for co‐expression analysis in *Nicotiana benthamiana*

Constructs of *35S::SPT‐RFP* and *35S::NLS‐RFP* were created as follows: the full‐length CDSs of *SPT* and *NLS* were digested with SifI (NEB) and cloned into the empty *pCambia1305–35S::RFP* vector, respectively, which was pre‐digested with DraIII (NEB). For the genomic *CYCP3;1* and *CYCP3;2* constructs (without STOP codon), Golden Gate cloning assembly was used as described above. The *CYCP3;1* promoter (2949 bp upstream of the start codon) and *CYCP3;2* (2845 bp upstream of the start codon) were cloned as described above. Genomic fragments of CYCP3;1 and CYCP3;2 transcript regions without the stop codon were amplified and cloned into pAGM1287. These entry clones were combined with CITRINE (Cit) in pAGM1301 and inserted into the L1 vector pICH47742 and finally into the destination vector pAGM4723 together with pICSL11059 that confers the Hygromycin resistance cassette. For the generation of the constructs harbouring different G‐BOX mutations of the promoters, p*CYCP3;1:CYCP3;1‐CITRINE* and p*CYCP3;2:CYCP3;2‐CITRINE* in pAGM4723 were used as templates, respectively, to introduce specific point mutations in the promoter sequence with mutagenesis primers (listed in Table S2). These binary constructs were introduced into *A. tumefaciens GV3101* strain for infiltration in *N. benthamiana* leaves.

### Chromatin immunoprecipitation sequencing (ChIP‐seq)

Young inflorescences were chopped off from 4 weeks old plants of *spt‐12/SPT*::*SPT*–*sYFP* line (Jiang et al., 2024) and immediately frozen in liquid nitrogen after collection as described previously (Jiang et al., 2024). 3 g of inflorescences were used for each biological replicate; the experiment was performed with 3 biological replicates. Collected tissue samples were cross‐linked in 1% formaldehyde under vacuum (3 × 5 min) and the reaction was quenched with 125 mM glycine for 5 min under vacuum. Samples were subsequently washed with PBS and water, blotted dry, wrapped in foil and flash‐frozen in liquid nitrogen as described previously (Kuhn & Ostergaard, 2020). Immunoprecipitation (IP) was conducted using GFP‐Trap^®^ magnetic particles M‐270 (ChromoTek, Planegg‐Martinsried, Germany). IP and input samples (*n* = 6, three for each IP and input) were sequenced by Novogene Illumina Sequencing (PE150). Raw reads were processed and trimmed using fastp (v0.20.1) and aligned to the TAIR10 genome with Bowtie2 (v2.5.1) (Langmead & Salzberg, 2012). The mapped data was sorted, indexed; duplicate reads were flagged using Samtools (Li et al., 2009) (v1.9). Reads overlapping blacklisted genome regions, as defined by the Greenscreen Project (Klasfeld et al., 2022), were removed using Bedtools (Quinlan & Hall, 2010) (v2.31.0). Peaks were called using MACS3 (v3.0.0a7) (Zhang et al., 2008) with the parameters callpeak ‐p 0.1 ‐B ‐‐bdg ‐‐keep‐dup auto. The resulting peaks were further filtered based on different cutoffs and submitted to PAVIS (Huang et al., 2013) for annotation and visualisation (https://manticore.niehs.nih.gov/pavis2/annotate). Peaks with a 0.001 FDR were assigned to gene models within 2 kb upstream and 1.5 kb downstream regions. Target genes were shortlisted if they appeared in all three replicates. ChIP‐seq data was visualised using the Integrative Genomics Viewer (Robinson et al., 2011). The peaks and the genome tracks were plotted using pyGenomeTracks (Lopez‐Delisle et al., 2021; Ramírez et al., 2018). The GO enrichment and visualisation was done using ShinyGO (Ge et al., 2020) v0.80 (bioinformatics.sdstate.edu/go) with FDR cut‐off 0.05 against *Arabidopsis thaliana* TAIR10 assembly.

### Bioinformatic analysis of ChIP‐seq_ and RNA‐seq_intersection of SPT targets

We downloaded the supplementary gene list from Bernal‐Gallardo et al. (2023) (Bernal‐Gallardo et al., 2023). To identify overlapping targets, our ChIP‐seq enriched SPT genes were compared with the RNA‐Seq–derived SPT gene set from this study (*n* = 3692). The overlapping genes (TAIR10 IDs) are provided in the Table S1 [Excel sheet chipseq_rnaseq_intersection‐spt‐targets‐ath.tab.xls]. The gene descriptions and their associated metadata were retrieved from (https://bar.utoronto.ca/thalemine/bag.do) (Krishnakumar et al., 2015; Pasha et al., 2020) and incorporated into the output. The scripts used to identify the intersection are available at https://github.com/gitbackspacer/spatulachipseq.

### ChIP‐qPCR assay

Following ChIP protocol as described above, the enrichment of CYCP3;1, CYCP3;2 and CYCD1;1 promoter regions (around the G‐boxes cis‐elements) was quantified using qPCR. Enrichment values were normalised against the *ACTIN7* gene. Primers used for the enrichment of each gene are listed in Table S2.

### RNA extraction and qRT‐PCR

For qRT‐PCR analysis, RNeasy Plant Mini Kit (Qiagen) was used to extract total RNA from either young inflorescences or 7 days old seedlings in triplicate (three independent biological replicates). Reverse transcription of extracted RNA was done using M‐MLV Reverse Transcriptase (Promega, Madison/Fitchburg, WI, USA). At least three independent technical experiments were performed from each RNA sample using SYBR Green Master Mix (Promega) with Chromo4 Real‐Time PCR Detection System (Bio‑Rad Laboratories, Hercules, CA, USA). Target gene expression levels were normalised against *UBIQUITIN10*. Relative expression levels were quantified using the 2^−ΔΔCT^ method in Microsoft Excel (v.2311). The results from one representative experiment are plotted in figures. The gene specific primers used for the analysis are listed in Table S2.

### Scanning electron microscopy

Whole inflorescences were fixed overnight in FAA (3.7% formaldehyde, 5% glacial acetic acid, 50% ethanol) and dehydrated through an ethanol series (50–100%) as described previously (Carabelli et al., 2021). After complete dehydration, samples were critical point‐dried using Leica EM CPD300. Gynoecia were dissected manually using a stereomicroscope (Leica S9D) and mounted on stubs. Samples were sputter coated using ACE‐600 before examination using an FEI Nova NanoSEM 450 emission scanning electron microscope. An acceleration voltage of 3 kV was used for imaging samples. The total number (*n*) of gynoecia imaged for each experiment is mentioned in the figures' legends.

### GUS histochemical analysis

To analyse *pCYCP3;1:GUS* and *pCYCP3;2:GUS* lines expression in wild‐type (Col‐0) and mutant/overexpression (*spt‐12*, *ind‐2*, *35S::ARR1ΔDDK:GR*) backgrounds, whole inflorescences were chopped off plants and acetone pre‐treatment and GUS staining were performed as described previously (Carabelli et al., 2021). For expression analysis of *pCYCP3;1:GUS* (T2), *spt‐12 pCYCP3;1:GUS* (F2), *ind‐2 pCYCP3;1:GUS* (F2) and *35S::ARR1ΔDDK:GRxpCYCP3;1:GUS* (F1), whole inflorescences were stained for 4.5 h. While, for visualisation of *pCYCP3;2:GUS* (T2), *spt‐12 pCYCP3;2:GUS* (F2), *ind‐2 pCYCP3;2:GUS* (F2) and *35S::ARR1ΔDDK:GRxpCYCP3xpCYCP3;2:GUS* (F1), staining was performed overnight (24 h). Likewise, overnight staining was performed to analyse expression of *pCYCP3;2:GUS* (T2) in ectopic outgrowths from ovary, while the staining duration of *pCYCP3;1:GUS* (T2) flowers was 5 h. Samples were washed with sterile water after decanting GUS‐solution and replaced with a few dilutions of 70% ethanol until chlorophyll was completely removed. Gynoecia were manually dissected using a stereomicroscope (Leica S9D) and mounted on glass slides in an 8:3:1 chloralhydrate (Merck) solution. Mounted samples were analysed by Zeiss Axio Imager Z2 light microscope using DIC prisms.

### Transient expression assay in tobacco leaves

The transient co‐expression assays were performed on 4‐week‐old *N. benthamiana* plants infiltrated with *A. tumefaciens* strains carrying the respective binary expression plasmids. *Agrobacterium tumefaciens* suspensions were prepared in infiltration buffer (10 mM MES, 10 mM MgCl_2_ and 150 μM acetosyringone, pH 5.6) and were adjusted to appropriate OD_600_. *Agrobacterium* strain harbouring P19 was also co‐infiltrated to enhance gene expression. After 48 h of infiltration, the infiltrated tobacco leaves were observed under the confocal laser‐scanning microscope.

### Confocal microscopy

For confocal imaging of tobacco leaves, a Zeiss LSM 880 confocal scanning microscope was used with the following fluorescence excitation–emission settings to visualise: CITRINE (*Cit*) excitation 514 nm, emission 530 nm; RFP excitation 550 nm, emission 580 nm. Pictures were taken with 20× or 40× water/oil immersion objectives. Samples within one experiment were imaged with identical settings. For image analyses, ImageJ and Zeiss Zen 2011 (v3.4) image analysis software were used.

### Statistical analysis

Relative expression levels were compared using Student's unpaired *t*‐test. *P* value <0.05 was considered significant. For comparison of phenotypic classes, 2 × 2 contingency tables were generated, followed by Fisher's exact Chi square test. Two‐tailed *P* values <0.0001 were considered extremely statistically significant. Experimental data was obtained by counting the number of phenotypes, while their percentage is plotted in the graphs. For style length and width comparison, one‐way anova (analysis of variance) followed by Tukey's Honestly Significant Difference (HSD) was used for pairwise comparisons. Tukey's HSD *P* values <0.001 were considered extremely statistically significant. Photoshop^®^ was used to assemble the figures.

## AUTHOR CONTRIBUTIONS

LM, conceptualised the project; LM and IJ designed the experimental research; IJ, performed most of the experimental work with help from SWHK, JC and LM. All authors analysed the data. LM prepared the figures and wrote the manuscript. All authors commented and edited the manuscript.

## CONFLICT OF INTEREST

The authors declare no competing interests.

## Supporting information


**Figure S1.** Presumptive direct targets of SPT from ChIP‐seq experiments.
**Figure S2.** Expression of *CYCP3s* in wild‐type (Col‐0), various mutant backgrounds and after CK treatments.
**Figure S3.** Cloning strategy for *CYCP3s* CRISPR mutants and expression levels of *CYCP3s* overexpressing lines.
**Figure S4.** Plant architecture of wild‐type (Col‐0), overexpression and loss‐of function mutants of CYCP3;1 and CYCP3;2.
**Table S2.** List of primers used in the study.

**Table S1.** Analysis of the *spt‐12/SPT*::*SPT*–*sYFP* ChIP‐seq experiments conducted in triplicates using inflorescent material, list of cell division genes and common genes between ChIP‐seq (this work) and RNA‐seq experiments (Bernal‐Gallardo et al., 2023).

## Data Availability

The *spt‐12/SPT*::*SPT*–*sYFP* raw ChIP‐seq datasets are available via EBI/NCBI website under study accession number PRJEB80813 (https://www.ncbi.nlm.nih.gov/bioproject/?term=PRJEB80813). All processed data are contained in the manuscript or in Supporting Information S1. Biological material and data from this study will be available upon request and with no restrictions.
