# Supplementary material for: Cytokinin‐responsive P‐type cyclins control Arabidopsis radial style morphology
Source: Plant J. 2025 Nov 24;124(4):e70592. doi: 10.1111/tpj.70592 (PMC12643530; doi:10.1111/tpj.70592)
Supplement: Supplementary file 1 — Figure S1. Presumptive direct targets of SPT from ChIP‐seq experiments. Figure S2. Expression of CYCP3s in wild‐type (Col‐0), various mutant backgrounds and after CK treatments. Figure S3. Cloning strategy for CYCP3s CRISPR mutants and expression levels of CYCP3s overexpressing lines. Figure S4. Plant architecture of wild‐type (Col‐0), overexpression and loss‐of function mutants of CYCP3;1 and CYCP3;2. Table S2. List of primers used in the study. [file TPJ-124-0-s001.docx]

**SUPPLEMENTARY MATERIAL**

**Cytokinin-responsive P-type CYCLINS control Arabidopsis radial style morphology**

Iqra Jamil^1^, Samuel. W.H. Koh^1,2^, Jitender Cheema^3,4^, and Laila Moubayidin^1,5,*^

1: Department of Cell and Developmental Biology, John Innes Centre, Colney Lane NR4 7UH, Norwich, Norfolk, United Kingdom.

2: current affiliation: Laboratory of Biochemistry, Wageningen University, Stippeneng 4, Wageningen, 6708WE, The Netherlands

3: Department of Computational and Systems Biology, John Innes Centre, Colney Lane NR4 7UH, Norwich, Norfolk, United Kingdom.

4: current affiliation: European Molecular Biology Laboratory, European Bioinformatics Institute, Wellcome Genome Campus, Hinxton, Cambridge, Cambridgeshire CB10 1SD, United Kingdom.

5: current affiliation: Dipartimento di Scienze Chimiche, della Vita e della Sostenibilità Ambientale, Università di Parma, Parco Area delle Scienze 11/A, 43124 Parma, Italy.

* email: Laila.moubayidin@unipr.it

**
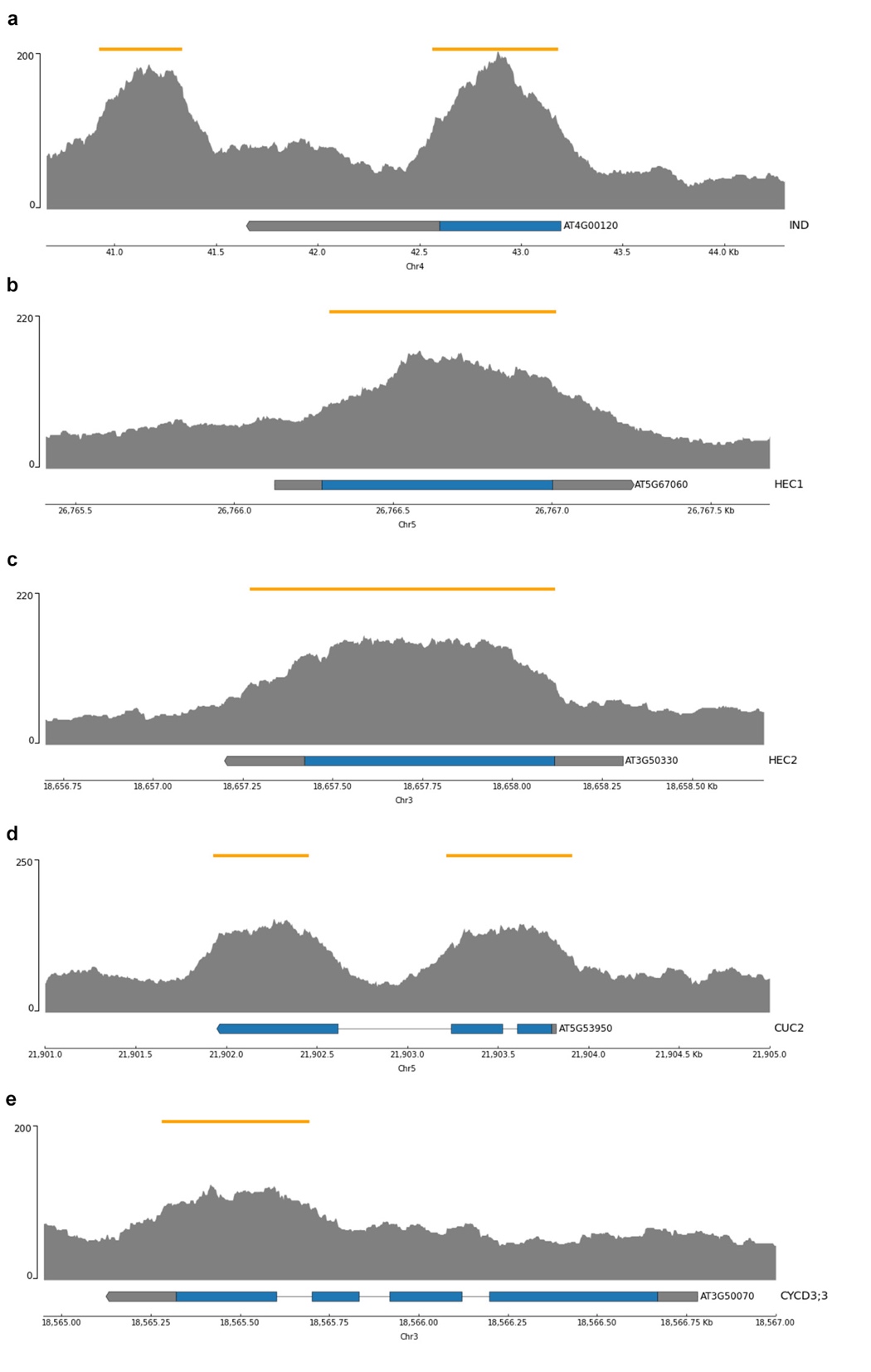
**

**Supplementary Fig.1. Presumptive direct targets of SPT from ChIP-seq experiments**

**a-e** Representative raw Chromatin Immunoprecipitation sequencing (ChIP-seq) peaks of *IND* (a), *HEC1* (b), *HEC2* (c), *CUC2* (d) and *CYCD3;3* (e). n=3 biological replicates; peaks of one representative replicate are shown. Yellow bars on top represent peaks position on chromosome. Blue bars on bottom show exons, and grey lines show introns.


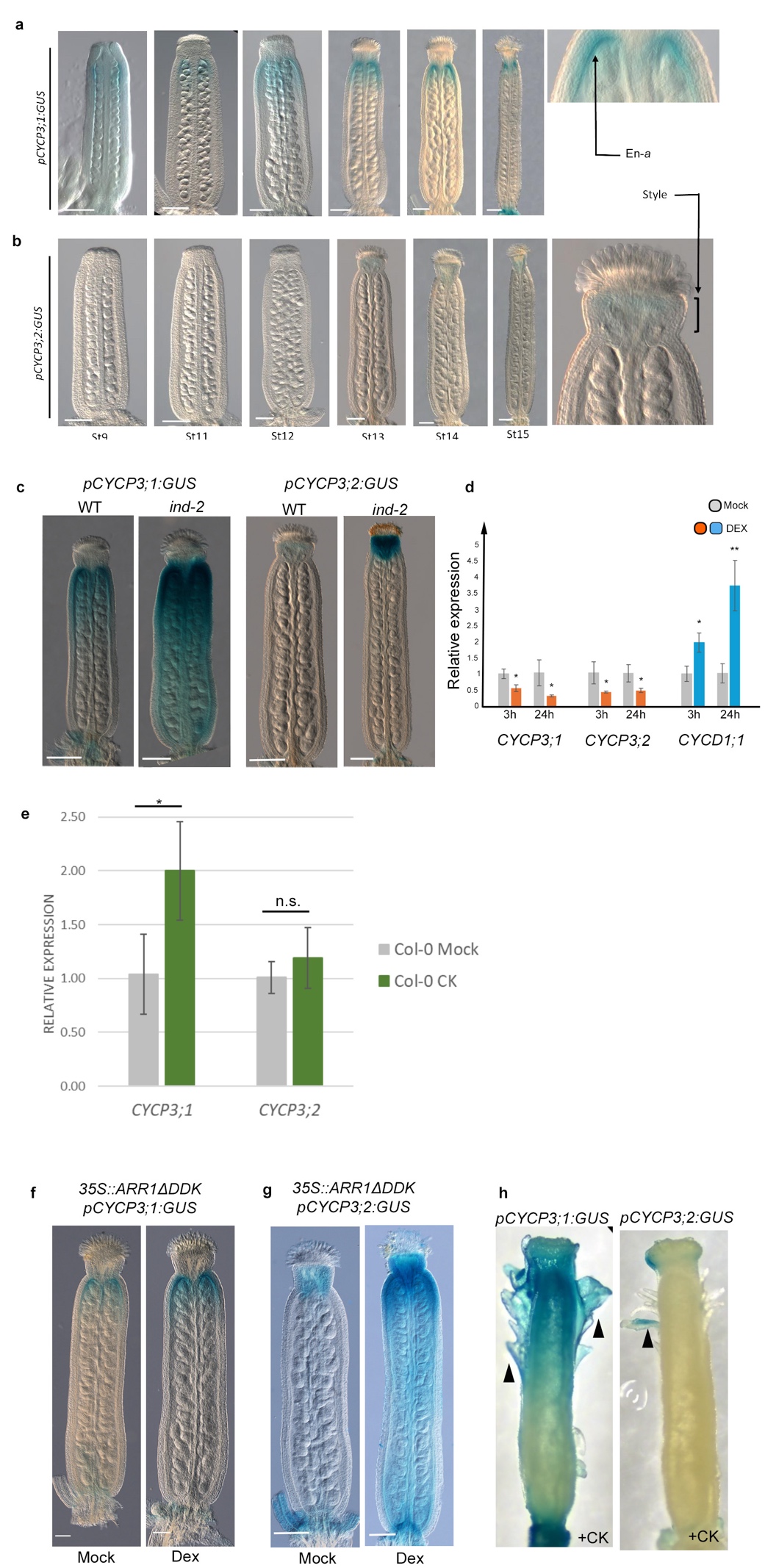


**Supplementary Fig.2. Expression of *CYCP3s* in wildtype (Col-0), variuos *mutant* backgrounds and after CK treatments.**

**a, b** Light microscope images of GUS-stained gynoecia (stages 9-15) showing expression of *pCYCP3;1:GUS* (a) and *pCYCP3;2:GUS* (b) in wildtype (Col-0) background. Notice the expression of *pCYCP3;1:GUS* in endocarp-*a* (En-*a*) throughout developmental stages, and a very weak signal of *pCYCP3;2:GUS* in style of stage-15 gynoecia. Scale bars are 100 µm. 50 gynoecia were analysed for each transgenic line. n=3 biological repetitions. **c,** Light microscope images of GUS-stained gynoecia of p*CYCP3;1:GUS* and p*CYCP3;2:GUS* in wildtype Col-0 and *ind-2* mutant backgrounds. Scale bars represent 200 μm. n=3 biological replicates and ~25-30 gynoecia were analysed for each genotype. **d,** Bar chart of qRT-PCR of *CYCP3;1*, *CYCP3;2* (orange bars) and *CYCD1;1* (blue bars) expression levels after 3h and 24h of DEX induction (orange and blue bars) of *35S::IND:GR* line compared to Mock (grey bars), normalised against *UBIQUITIN10*. n=3 biological replicates with 10 seedlings per replicate. Error bars represent SD; *p<0.05, **p<0.001 (unpaired Student’s *t*-test). **e,** Bar chart of qRT-PCR analysis of *CYCP3s* expression in WT (Col-0) flowers treated with cytokinin (CK) for 2 consecutive days compared to mock treated, normalised against *UBIQUITIN10*. n= 3 replicates with 5-6 flowers per replicate. (Student’s t test *p=0.05, ns=non significant). Error bars represent SD. **f** and **g,** Light microscope images of GUS-stained mock- (left) and DEX- (right) treated (10 µM) gynoecia of F1 *pCYCP3;1:GUS x 35S::ARR1ΔDDK:GR* (f) and *pCYCP3;2:GUS* (g) *x 35S::ARR1ΔDDK:GR.* 20 to 40 gynoecia were analysed for each F1 cross. n=3 biological repetitions. Scale bars are 200 µm. **h,** Stereo microscope images of GUS-stained gynoecia showing expression of *pCYCP3;1:GUS* and *pCYCP3;2:GUS* in medial outgrowths from ovary upon CK treatment. 30 gynoecia were analysed for each transgenic line. n=3 biological replicates.

**
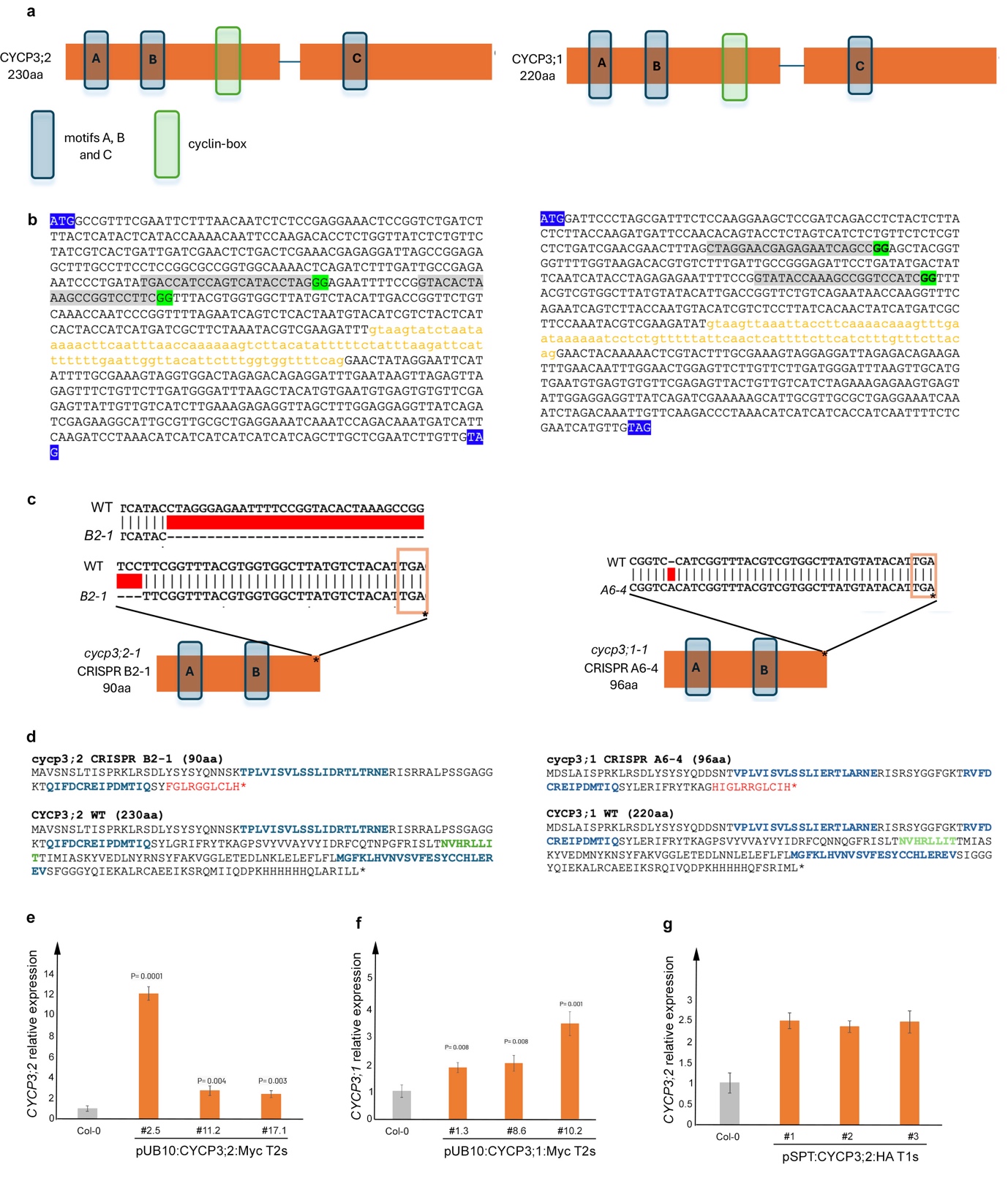
**

**Supplementary Fig.3. Cloning strategy for *CYCP3s* CRISPR mutants and expression levels of *CYCP3s* overexpressing lines.**

**a,** Schematic representation of CYCP3;2 (left) and CYCP3;1 (right) protein structures showing exons (orange boxes), intron (blue line), the A,B,C motifs (blue boxes) and the cyclin-box protein domain (green box). **b**, Genomic nucleotide protein sequence of CYCP3;2 (left) and CYCP3;1 (right) showing the position of the two guides (highlighted in grey) used to obtain the single *cycp3;2-1* and *cycp3;1-1* CRISPR mutants. The PAM recognition site (GG) is highlighted in green, the start and stop codons are highlighted in blue, exons are represented by black font while the intron is in orange font. **c**, Schematic representation of CYCP3;2 (left) and CYCP3;1 (right) CRISPR products obtained by DNA sequencing of T2 plants. The scheme includes the alignment of WT and mutated sequences and shows the position of the big deletion recovered in *cycp3;2-1* (line #B2-1) and of a single-nt (nucleotide) insertion in *cycp3;1-1* (line #A6-4). The shift of the open reading frame in both cases generated a premature STOP codon (TGA) in the first exon after the A and B motifs and before the cyclin-box domain. **d**, Prediction of the amino acid sequence of *cycp3;2-1*(90 aa, left) and *cycp3;1-1* (96 aa, right) generated by CRISPR-Cas9 compared to the respective wild-type sequences (230 aa for CYCP3;2 and 220 aa for CYCP3;1), showing the position of the A and B motifs (blue font) and the shifted open reading frame (red font) generated by the mutations. Asterisks represent STOP codons. **e, f** Bar charts of qRT-PCR quantification showing relative expression of *CYCP3;2* (e) and *CYCP3;1* (f) in wildtype (Col-0) background compared to three independent overexpressing homozygous lines (T2s) of *UB10::CYCP3;2:Myc* (#2.5, #11.2 and #17.1) and *UB10::CYCP3;1:Myc* (#1.3, #8.6 and #10.2). Expression levels were normalised against *UBIQUITIN10*. n= 3 biological replicates. Error bars represent SD. Statistical significance, *p* values (Student’s *t-*test) are shown on each bar. **g** Bar charts of qRT-PCR quantification showing relative expression levels of *CYCP3;2* in Col-0 compared to three independent transgenic lines (T1s) of *pSPT:CYCP3;2:HA*. Expressions were normalised against *UBIQUITIN10*. The experiment was performed once on three independent transgenic lines, with four technical repeats. Values shown are means ± SEM.


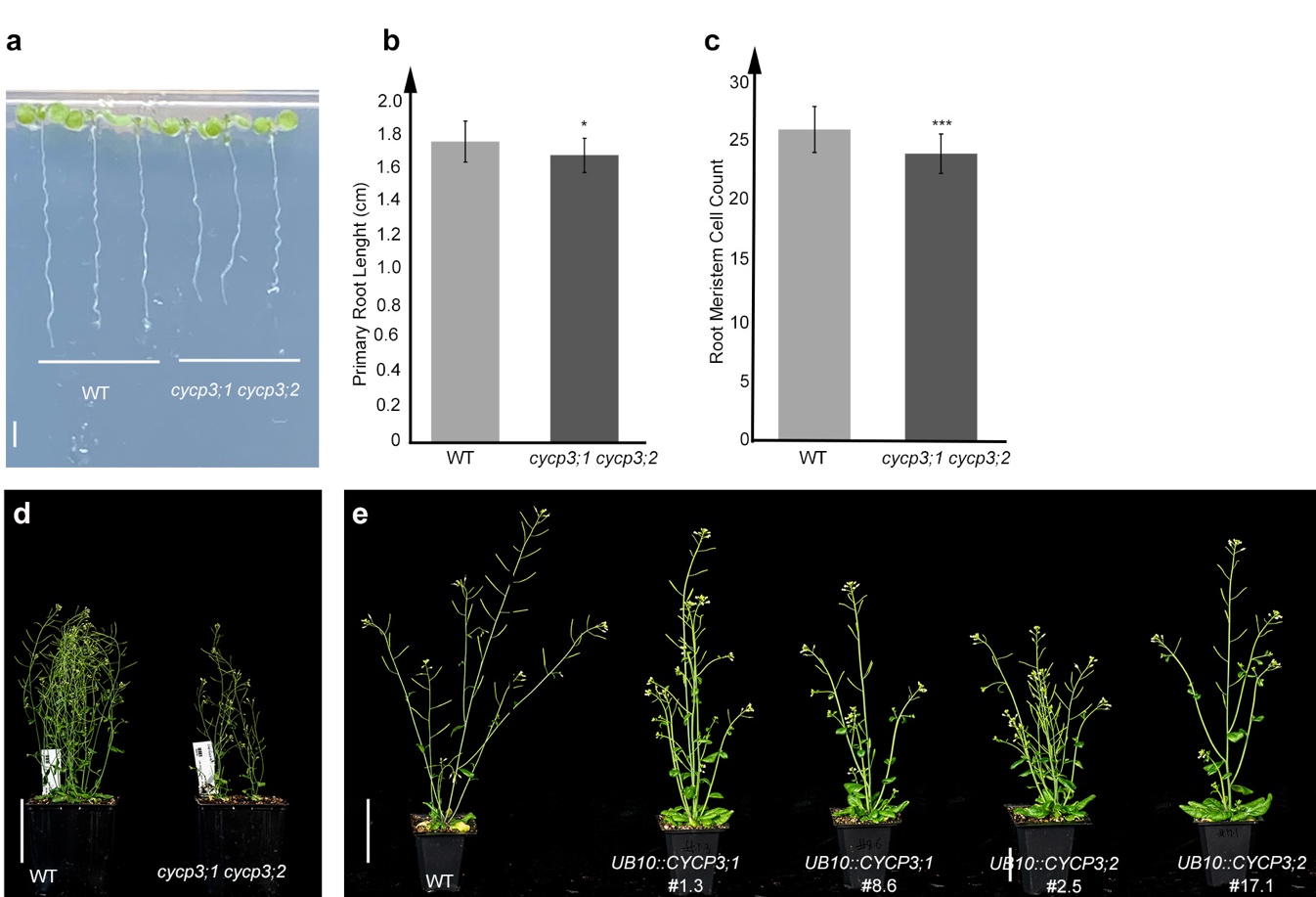


**Supplementary Fig.4. Plant architecture of wildtype (Col-0), overexpression and loss-of function mutants of CYCP3;1 and CYCP3;2.**

**a,** 7 days old seedlings of WT (Col-0) and loss-of function double mutant *cycp3;1 cycp3;2*. Scale bar represents 200μm. **b,** Measurement of primary root length (cm) of WT (Col-0) and *cycp3;1 cycp3;2* double mutant. **c**, Measurement of number of cortical cells in the root meristem of WT (Col-0) and *cycp3;1 cycp3;2* double mutant. Results from one of three representative biological replicates are shown here, with 30 seedlings analyzed per genotype. Error bars represent SD; *p<0.05, ***p<0.0001 (unpaired Student’s *t*-test). **d,** Five-week old plants of WT (Col-0) and *cycp3; 1 cycp3;2* double mutant. All plants were grown in controlled environment room (CER) in standard conditions. Scale bar represents 5cm. **e,** Four-week old plants of WT (Col-0), and 2 independent homozygous T2 lines of *pUB10:CYCP3;1:Myc* (#1.3, #8.6) and *pUB10:CYCP3;2:Myc* (#2.5, #17.1). Scale bar represents 5cm.

Data Table S1 (separate excel file)

Analysis of the *spt-12/SPT*::*SPT*–*sYFP* ChIP-seq experiments conducted in triplicates using inflorescent material, list of cell division genes, and common genes between ChIP-seq (this work) and RNA-seq experiments [26].

Data Table S2

List of primers used in the study

| Primer | Sequence (5'-3') | Purpose |
| --- | --- | --- |
| CYCP3;1_gORF_F | GGAAGACGGAATGGATTCCCTAGCGATTTCTCC | Cloning strategy for genomic Open Reading Frame of CYCP3;1 and CYCP3;2 |
| CYCP3;1_gORF_R | GGAAGACGGCGAACAACATGATTCGAGAAAATTGATGG |  |
| CYCP3;2_gORF_F | GGAAGACGGAATGGCCGTTTCGAATTCTTTAAC |  |
| CYCP3;2_gORF_R | GGAAGACGGCGAACAACAAGATTCGAGCAAGCTG |  |
| CYCP3;2_F (KpnI) | CGGGGGACTCTAGGGGTACCATGGCCGTTTCGAATTCTTTAAC | clone CYCP3;2 coding sequence to *pCambia1305* |
| CYCP3;2_R (XhoI) | TAGAGGATCCCTCGAGCAACAAGATTCGAGCAAGCTG |  |
| IPT7_F(KpnI) | CGGGGGACTCTAGGGGTACCATGAAGTTCTCAATCTCATCACTGA | clone IPT7 coding sequence to *pCambia1305* |
| IPT7_R (XhoI) | TAGAGGATCCCTCGAGTCATATCATATTGTGGGCTCTACT |  |
| Cycp3;2_guide9_F | TGTGGTCTCAATTGTGACCATCCAGTCATACCTAGTTTAAGAGCTATGCTGGAA | Cloning strategy for CRISPR CYCP3;1 and CYCP3;2 mutants |
| Cycp3;2_guide6_F | TGTGGTCTCAATTGGTACACTAAAGCCGGTCCTTGTTTAAGAGCTATGCTGGAA |  |
| Cycp3;1_guide3_F | TGTGGTCTCAATTGCTAGGAACGAGAGAATCAGCGTTTAAGAGCTATGCTGGAA |  |
| Cycp3;1_guide1_F | TGTGGTCTCAATTGGTATACCAAAGCCGGTCCATGTTTAAGAGCTATGCTGGAA |  |
| REV_univ | TGTGGTCTCA AGCG AAAAAAAGCACCGACTC |  |
| CYCP3;1_CRISPRseq_F | GAACTTTCGTAGTTTGCACG |  |
| CYCP3;1_CRISPRseq_R | CACACTCACATTCACATGCAAC |  |
| CYCP3;2_CRISPRseq_F | AACGCAATGCCTTCTCGATC |  |
| CYCP3;2_CRISPRseq_R | AACGCAATGCCTTCTCGATC |  |
| Spt-12 LP | TGACTTGGAAGAGGGAGCTTCA | genotyping of *spt-12* mutant |
| SPT-12 RP | GAAGAAGCAGAGAGTGATGGGAGA |  |
| SPT-12 LB | AACGTCCGCAATGTGTTATTAAGTTGTC |  |
| pCYCP3;1_F | GGAAGACGGGGAGggtccacatcattgtttccatatg | Cloning strategy for *pCYCP3;1:GUS* and *pCYCP3;2:GUS* |
| pCYCP3;1_R | GGAAGACGGCATTtgatcggagaaactttatgggag |  |
| pCYCP3;2_F | GGAAGACGGGGAGggagcgaagcatatatgtatacg |  |
| pCYCP3;2_R | GGAAGACGGCATTtgatgtttcgccggaaagtac |  |
| ACTIN7-F | GATATTCAGCCACTTGTCTGTG | CHIP-qPCR |
| ACTIN7-R | CTTACACATGTACAACAAAGAAGG | CHIP-qPCR |
| pCYCP3;1 CHIP4-F | TTCGAAACTCACAACGCTCG | CHIP-qPCR |
| pCYCP3;1 CHIP4-R | AAACAAAGAAGACTCCGCCG | CHIP-qPCR |
| pCYCP3;2 CHIP3-F | CATGATGAAGTAACCGCACGT | CHIP-qPCR |
| pCYCP3;2 CHIP3-R | CCAAACTAAGAATCGCCGCC | CHIP-qPCR |
| CYCD1;1 CHIP6-F | GGAGATGAATCAAACCGGAGC | CHIP-qPCR |
| CYCD1;1 CHIP6-R | ACCTTCACTCTTCTCTCCACA | CHIP-qPCR |
| IPT 7 3'UTR-F | CGAGACCAAGCGAGAGAATC | qRT-PCR |
| IPT 7 3'UTR -R | TCTACTTCCACTTCCTCCGC | qRT-PCR |
| UBIQUITIN10-F | GGCGTTGTATAATCCCTGATGAATAAG | qRT-PCR |
| UBIQUITIN10-R | AAAGAGATAAACAGGAACGGAAACATAGT | qRT-PCR |
| HA tag-R | CTGGAACGTCATATGGATAGG | qRT-PCR |
| CYCP3;2 2^nd^ exon-F | AGATGGACTAGAGACAGAGGA | qRT-PCR |
| CYCP3;2 2^nd^ exon-R | AACGCAATGCCTTCTCGATC | qRT-PCR |
| CYCP3;1 3'UTR-F | TGCATGTGAATGTGAGTGTGT | qRT-PCR |
| CYCP3;1 3'UTR-F | CGTGGACTACAACATGATTCGA | qRT-PCR |
| Myc tag-R | CCTCCTCTGAGATAAGCTTCTGCTC | qRT-PCR |
